# Zymophore identification enables the discovery of novel phenylalanine ammonia lyase enzymes

**DOI:** 10.1038/s41598-017-13990-0

**Published:** 2017-10-20

**Authors:** Nicholas J. Weise, Syed T. Ahmed, Fabio Parmeggiani, James L. Galman, Mark S. Dunstan, Simon J. Charnock, David Leys, Nicholas J. Turner

**Affiliations:** 1 School of Chemistry, Manchester Institute of Biotechnology, University of Manchester, 131 Princess Street, Manchester, M1 7DN United Kingdom; 2 SYNBIOCHEM, Manchester Institute of Biotechnology, University of Manchester, 131 Princess Street, Manchester, M1 7DN United Kingdom; 3 Prozomix Ltd., Station Court, Haltwhistle, Northumberland, NE49 9HN United Kingdom

## Abstract

The suite of biological catalysts found in Nature has the potential to contribute immensely to scientific advancements, ranging from industrial biotechnology to innovations in bioenergy and medical intervention. The endeavour to obtain a catalyst of choice is, however, wrought with challenges. Herein we report the design of a structure-based annotation system for the identification of functionally similar enzymes from diverse sequence backgrounds. Focusing on an enzymatic activity with demonstrated synthetic and therapeutic relevance, five new phenylalanine ammonia lyase (PAL) enzymes were discovered and characterised with respect to their potential applications. The variation and novelty of various desirable traits seen in these previously uncharacterised enzymes demonstrates the importance of effective sequence annotation in unlocking the potential diversity that Nature provides in the search for tailored biological tools. This new method has commercial relevance as a strategy for assaying the ‘evolvability’ of certain enzyme features, thus streamlining and informing protein engineering efforts.

## Introduction

Currently, a major challenge in the area of enzyme discovery is the accurate retrieval from databases of enzymes with specific traits, from among the vast number of related sequences, an approach hindered by misannotation from sequence curation methods which are often implemented in the absence of specialist protein functional knowledge^1^. This problem is especially relevant in enzymes which are structurally diverse but have convergently evolved similar catalytic residue groupings, or in families where the function of choice constitutes an interspersed minority of available sequences. The former problem has been addressed in an enzyme class-specific manner by the computational assignment of clusters of active site residues conferring the desired chemistry on otherwise uncharacterised proteins. Examples include the mapping of active site ‘constellations’ for ene-reductase activity^2^, use of BioGPS descriptors to aid discovery of promiscuous side-activity^3^ or the retrieval of catalytic residue arrangements via the Catalytic Site Atlas or PatternQuery^4,5^. Whilst these methods provide simple means of accessing proteins with desired catalytic activity, their use is mostly limited to structural databases containing only a fraction of the number of entries that a sequence database would have. Such constraints are also less amenable to family-specific catalytic arrangements not documented elsewhere in nature where a wealth of sequence data can potentially harbour more scope for investigation. The structural methods also do not take into account other active site residues which differentiate desired and unwanted activity, such as selectivity and substrate discrimination.

With the ever diminishing cost and labour associated with DNA sequencing, cloning and synthesis, it is increasingly conceivable to imagine harnessing, from publicly available databases, the natural protein sequence diversity provided by evolution^6^. Such an approach offers several advantages over more traditional engineering methods, particularly for enzymes where robust high-throughput screening methodologies do not exist^7^. It also provides a means of mitigating changes deleterious to enzyme integrity whilst also allowing exploration of variation at non-active site positions, said to be important for contributions to activity^8^, and which are more numerous and thus difficult to target effectively.

**Figure 1:**
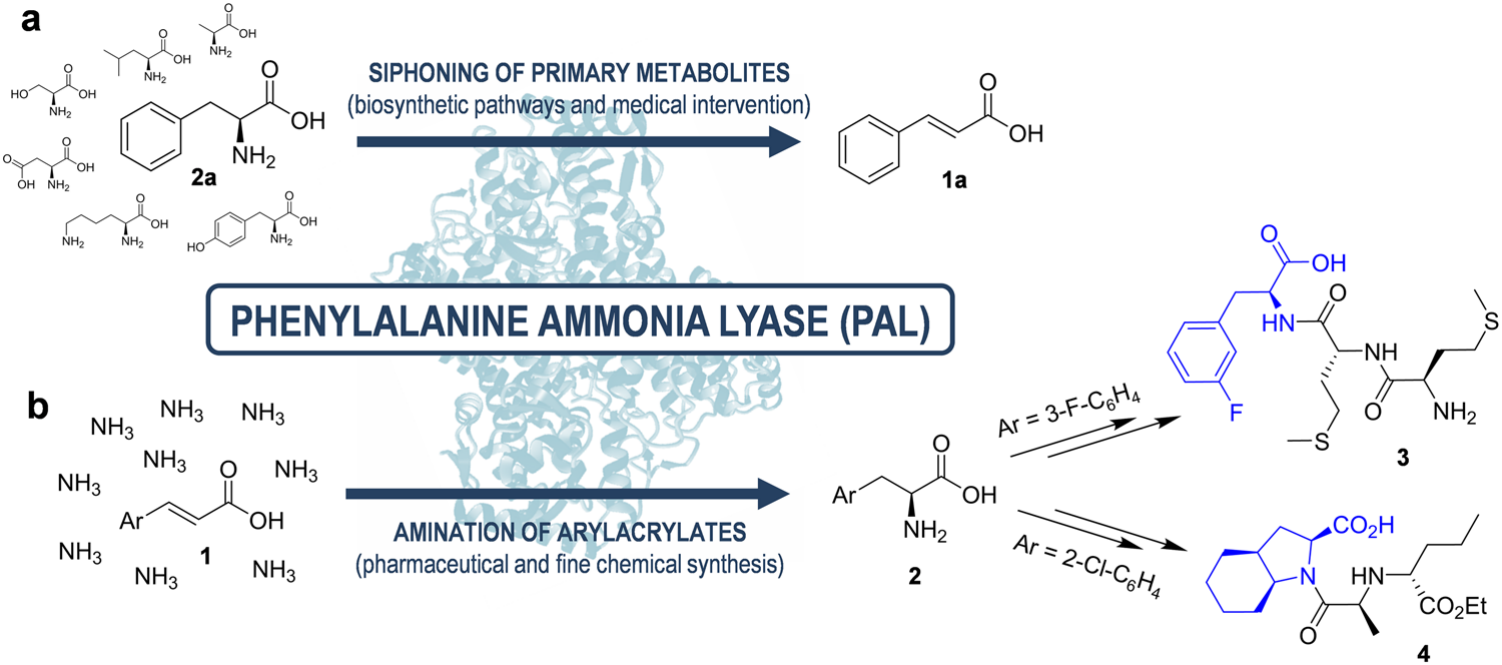
The catalytic activity of phenylalanine ammonia lyase (PAL) enzymes as implicated in biosynthesis/biotherapeutics (**a**) and biocatalysis (**b**).

Enzymes with phenylalanine ammonia lyase (PAL) activity are able to catalyse non-oxidative deamination to yield cinnamate (**1a**) and ammonia from the proteinogenic amino acid L-phenylalanine (L-Phe, **2a**)^9^. The amine abstraction, in this case, is mediated by adduct formation with a 4-methylideneimidazole-5-one (MIO) post-translational modification in the active site^10,11^. This in turn promotes elimination in conjunction with proton abstraction via a catalytic tyrosine residue situated on an inner active site mobile loop lid^11,12^. In nature, this PAL-mediated reaction often constitutes a gateway from primary metabolism to specialist compound biosynthesis, with examples including phenylpropanoids in plants^13^ and various antibiotics in bacteria (Fig. 1a)^14–17^.

In the field of biomedical research, the selective catabolism of L-Phe by PALs has been exploited as a treatment for a number of diseases. One such example is phenylketonuria, an inborn error of metabolism in which metabolic dysregulation leads to neurological symptoms such as microcephaly, seizures and intellectual disabilities^18^. As the condition emanates from aromatic amino acid imbalance - high phenylalanine and low tyrosine levels - removing L-Phe has been shown to offer great advantages over alternative treatments based on complex dietary restriction which are currently prescribed^19,20^. Essential amino acids, required by all growing cells, can also be degraded *in vivo* to restrict cancer tumour growth (amino acid depletion therapy), a promising strategy that has been demonstrated with metabolites such as L-Asn, L-Arg, L-Met, L-Tyr and aptly L-Phe^21^. Relevant examples with respect to the last example in particular include the decrease in metastatic phenotype of B16BL5 melanoma upon aromatic amino acid depletion^22^ and the use of PAL-based formulations to prevent growth of abnormal lymphocytes both *in vitro* ^23^ and in leukemic mice^24^. The commercial importance of PALs as therapeutic agents is evidenced by various patent applications from specialist enzyme (Codexis)^25^ and biopharmaceutical (BioMarin)^26^ production companies, as well as from multinational drug and chemical corporations (Novo Nordisk and BASF)^27,28^.

PALs also find use in the area of green chemistry and sustainable pharmaceutical manufacture as industrial biocatalysts, due to the reversibility of the deamination reaction (Fig. 1b). The use of concentrated yet inexpensive ammonia buffers has been demonstrated to allow regio- and enantioselective amination of a broad range of readily-accessible arylacrylic acids, yielding high value unnatural amino acids, a route that is difficult to emulate via chemical methods. Basic molecular cloning techniques have enabled the simple preparation of enzyme-based catalysts from renewable feedstocks, allowing the exploitation of their inherent selectivity and ambient reaction conditions by synthetic chemists. As such, there are many examples of biocatalytic routes in industry, including PAL-mediated processes to produce L-Phe^29^, unnatural amino acid building blocks of antifungal peptides (*e.g*., **3**)^30^, and the antihypertensive drug perindopril (**4**)^31,32^. The use of PALs for aromatic amino acid synthesis is particularly attractive due to the 100% atom efficiency and coenzyme independence of the reaction, mitigating waste production, by-product separation requirements and mediator supplementation/regeneration costs^30^.

In spite of the broad scope of application associated with PALs, their use is limited to a handful of specific enzymes^29,33,34^, greatly restricting the variability available in desirable traits. Recent efforts have focused on modification of the PAL from *Anabaena variabilis* (AvPAL), via mutagenesis or chemical conjugation, to overcome characteristics of the enzyme which are considered detrimental to therapeutic applications^19,20,25^. Such traits generally include low catalytic activity, poor stability at 37 °C, accessible proteolytic cleavage sites, low acid tolerance (if taken orally), aggregation and high immunogenicity. The few PALs used in a biocatalytic context (of which AvPAL is again the most prominent)^34–36^ show more immediate promise, with little or no engineering required for some industrial applications. However many pharmaceutically-relevant targets that are potentially within the remit of the synthetic PAL reaction remain out of reach due to the restricted substrate scope of characterised enzymes.

**Figure 2:**
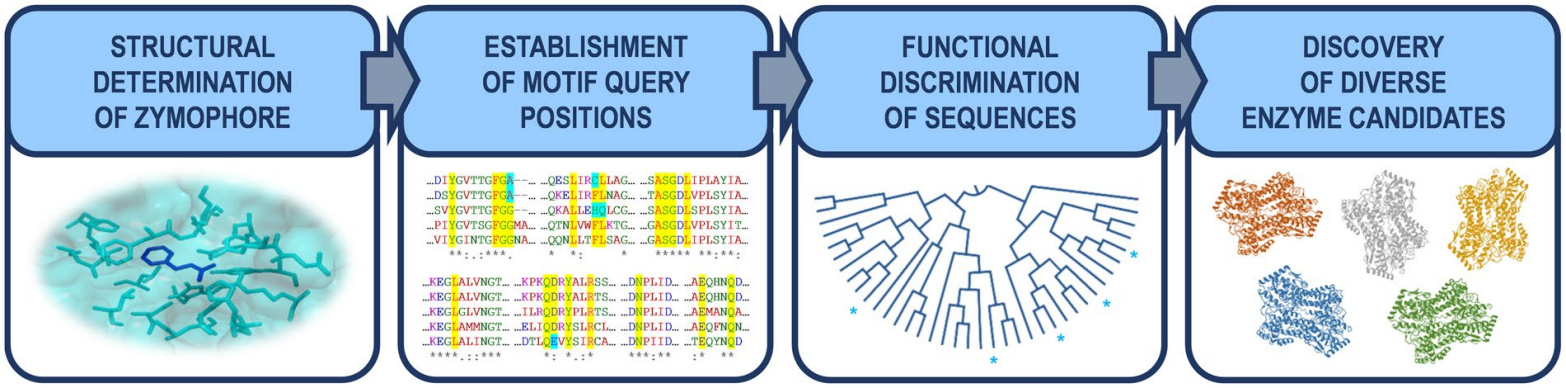
Flow diagram of the strategy employed to find new PAL enzymes for synthetic and medical applications.

In the class I lyase-like family, aminomutases and histidine/tyrosine-specific enzymes are reasoned to form the majority of available sequences, with PALs constituting the minority^37^. We therefore set out to develop a new strategy for the discovery of PAL enzymes based on the four-step process outlined in Fig. 2.

## Results and Discussion

Although several crystal structures of PAL enzymes have been solved, none exists with the natural ligand *trans*-cinnamic acid bound in the active site, thereby preventing correct and unambiguous assignment of the phenylalanine-specific MIO-dependent ammonia lyase zymophore. The gene encoding AvPAL was mutated, incorporating two surface substitutions (C503S and C565S, known to aid crystallisation^38^) and replacement of the loop tyrosine with a catalytically inert but shape-complementary phenylalanine (Y78F). The triple variant was purified and co-crystallised with the natural deamination product. Following successful crystal growth, the structure of the AvPAL-ligand complex (PDB ID: 5LTM) was solved by molecular replacement using the previously obtained ligand-free double variant (PDB ID: 3CZO) as a search model. Superimposition of these two structures revealed excellent complementarity with a few small differences between the unoccupied and occupied active sites. The only backbone displacement was a shift in the outer active site loop, with the fully closed conformation from the previously reported structure seen to clash with the inner active site position 78 in the co-crystallised protein. In the case of the unoccupied enzyme, the amino acid at position 78 is tyrosine which points inward toward the active site, providing no contact with the outer loop, whereas the new structure features Y78F with a dihedral swing of ~140° causing the bulky side chain to protrude outwards. Other differences included H359 moving into the active site sphere in the cinnamate bound structure, along with smaller shifts in the positions of Q311 and R317 (SI, Fig. S1). Presumably these represent movements which occur upon substrate binding, although they may be induced instead by the Y78F variation as exchange of polar and non-polar residues in the inner active site loop of related enzymes has been reported to influence conformational dynamics^39^. Interestingly, these three seemingly mobile positions have been identified as hotspots in mutagenic studies of various PAL-relatives, with variation at H359 shown to affect catalytic efficiency^36^ and the two carboxylate-binding positions (Q311 and R317) being implemented in substrate positioning and selectivity^40,41^. This observation sheds light on possible conformational selection or induced fit models which may help to guide engineering of enzymes in both this class and others by targeting residues which distinguish bound and unbound conformations.

**Figure 3:**
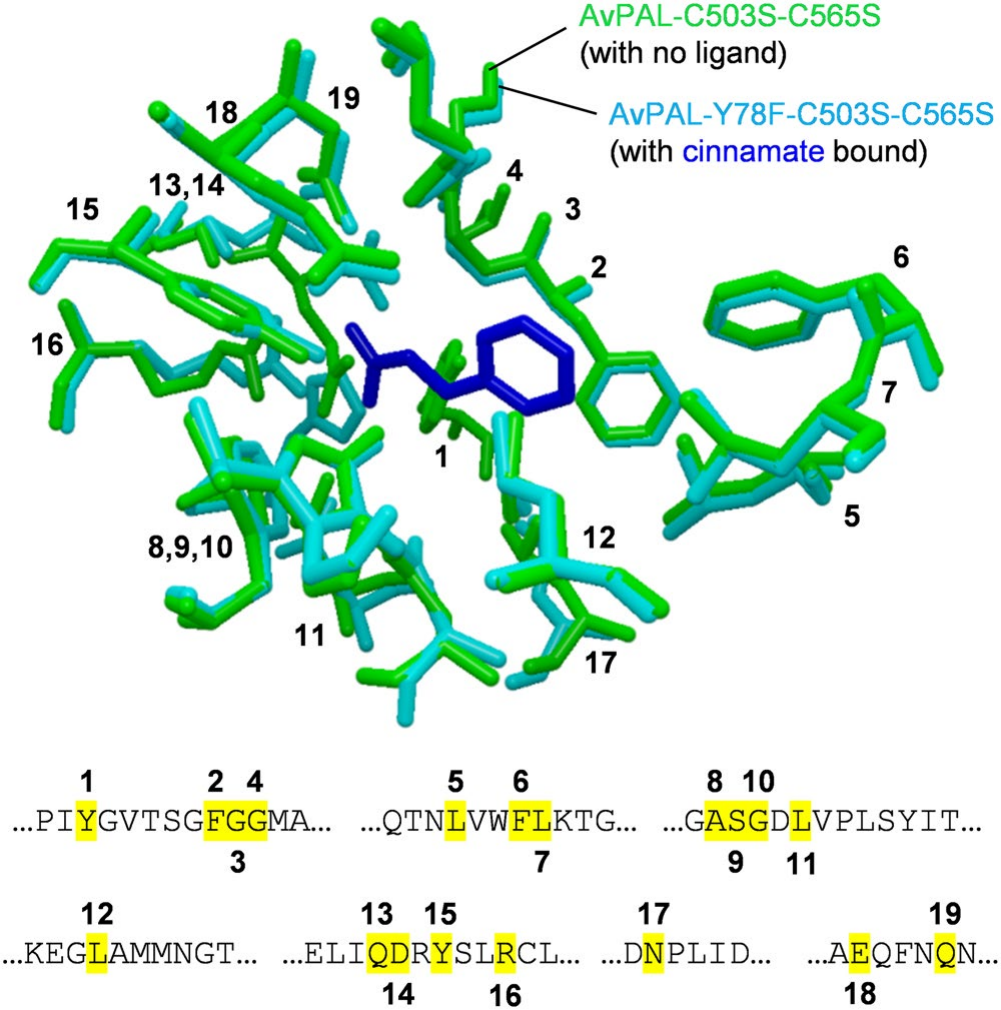
The zymophore conferring PAL-specific reactivity and selectivity as identified from the co-crystal structure of the ammonia lyase from *Anabaena variabilis* (AvPAL) and its deamination product *trans-*cinnamate (PDB ID: 5LTM). The motif is shown on both structural overlay of the empty and occupied enzyme active sites and the sequence with each of the 19 amino acids given a number to mark its position.

Visualisation of the occupied active site allowed the identification of 19 residues within a 6 Å sphere of the ligand (Fig. 3), including the MIO and catalytic amino acid at position 78, the carboxyl positioning residues, the hydrophobic aryl binding pocket and other assorted substrate-constraining side chains. As a test of the orthogonality of this PAL zymophore, a sequence alignment was performed using characterised ammonia lyase and aminomutase enzymes specific to phenylalanines and/or tyrosine or histidine (SI, Fig. S3). All the PALs in the selection showed conservation at all or all but one of the residues, while ammonia lyases accepting both L-Phe and L-Tyr (bifunctional PAL/TALs) and (*R*)-selective aminomutases ((*R*)-PAMs) varied at two positions. Bacterial lyases and mutases specific to tyrosine (TALs and TAMs) and histidine ammonia lyases (HALs) showed conservation at only 15 residues with (*S*)-β-Phe forming mutases ((*S*)-PAMs) scoring just 13/19. Interestingly, even those enzymes which differed by the same number of residues, tended to do so at different positions, highlighting the importance of using as many of the residues as possible in further investigations. As such, the entire motif was used and a score of 18/19 was deemed to be the threshold for consideration of sequences as potential PALs.

The 19 amino acid active-site sequence motif, as inferred from the AvPAL-cinnamate structure, was used to distinguish potentially useful enzyme sequences from other family members. The sequences were collected using the basic local alignment search tool (BLAST) to search the entire knowledge database of the universal protein resource (UniProtKB) using various query sequences.

As the most widely reported enzyme of this class in applied research, the protein sequence of AvPAL was used for the first search and the 100 hits showing the closest identity were analysed. Of these, 17 were shown to give close agreement with the desired active site, with the only differences occurring at positions 6 and 7 of the motif if at all. Four of the results, including the previously characterised PAL from *Nostoc punctiforme* (NpPAL)^42^, had hydrophobic residues at these positions, whereas all others contained a histidine at position 7. This variation is known to be associated with strict TAL activity in the bacterial enzyme BagA^43^. The 13 potential TALs were discounted along with NpPAL leaving a closely related sequence from *Oscillatoria* sp., and two more distant relatives from *Planctomyces brasiliensis* and *Methylobacterium* sp. Next, the sequence of the phylogenetically-distinct PAL, StlA from *Photorhabdus luminescens* ^16^, was used to search for PALs in a different part of the family tree. This time, within the first 100 hits the majority were found to contain YH or FH at positions 6 and 7, indicating possible unwanted TAL activity. Among the few PAL-like sequences, most were from the related enterobacterial genera *Photorhabdus* and *Yersinia* sharing high sequence identity with the query sequence. The remaining four PAL sequences were surprising in that they were from the amoebozoa genus *Dictyostelium*, whereas all other sequences were of eubacterial origin.

Lastly, the bacterial TAL sequence BagA from *Streptomyces* sp. was used for one final search to find PAL sequences not closely related to those currently known. It is worth noting that, although most of the hits were found to be TAL-like, with YH and FH at positions 6 and 7, there were more notable and diverse examples of active sites matching the AvPAL-derived motif than with either of the searches using characterised PAL query sequences. These included the previously reported RxPAL from *Rubrobacter xylophilans* ^44^, as well as additional sequences from *Bacillus subtilis* and *Actinopolyspora erythraea* and several from both *Brevibacillus laterosporus* and various species of the genus *Streptomyces*.

Interestingly the PALs from *Dictyostelium discoideum* and *Brevibacillus laterosporus* have been previously identified by Nielsen *et al*. using a synteny-based approach to search for class I lyase-like genes in the putative context of TAL-containing metabolic pathways^45^. This is an interesting finding, given the identification of these enzymes as PALs by their active sites in this study (rather than TALs based on inference of genetic co-localisation) and also given the rarity of PAL-like sequences seen in our database searches compared to the abundant TAL-like sequences. To add further evidence to the assertion that these sequences possessed PAL activity, genetic context analyses were conducted, revealing association with fatty acid and polyketide biosynthetic operons as has been reported previously with this class of enzyme^42^. In the case of *Brevibacillus laterosporus* the enzyme could even be putatively linked to the production of the cinnamate component of basiliskamides A and B, a link missing from previous biosynthetic hypotheses (SI, Fig. S7)^46^. Five of the potential PAL sequences mined from the protein database using the AvPAL zymophore motif were chosen for characterisation based on their sequence identity to their closest characterised relative and the results of the genetic context analyses (Table 1).

**Table 1:** The most prominent PAL sequences identified from UniProtKB.

Name	Origin	Motif match	Query	Seq. id.
BlPAL	*Brevibacillus laterosporus*	19/19	BagA	58%
SrPAL	*Streptomyces rimosus*	19/19	BagA	54%
MxPAL	*Methylobacterium* sp.	18/19	AvPAL	50%
DdPAL	*Dictyostelium discoideum*	18/19	StlA	48%
PbPAL	*Planctomyces brasiliensis*	19/19	AvPAL	46%

In all searches using characterised query sequences, evidence was uncovered of multiple PAL-TAL neofunctionalisation events within certain clades in the family, with indication of known PAL-active site motifs (with FL at selectivity positions) interspersed among known TAL motifs (with YH) and a possibly intermediary motif (FH). These findings point to the incidence of several substrate switching events resulting in distinct and unreported clades of TAL enzymes. This phenomenon may constitute an evolutionary strategy for the creation of chemical diversity in related biosynthetic pathways, interchanging between use of L-Phe or L-Tyr as a starting point, followed by further diversification and mixing of secondary metabolic enzymes to create new products. The searches also reveal that TAL-like sequences vastly outnumber PALs in the areas of sequence space targeted in this study, further demonstrating the importance of robust annotation methods over more random sampling of sequence diversity with this family of enzymes. The most interesting new sequences from the database were the PALs from *Dictyostelium* sp. including DdPAL, an example of an aromatic amino acid ammonia lyase from the protist kingdom. At around 50% sequence identity to StlA, this enzyme arises from a fairly recent gene transfer event between bacteria and eukaryotes, separate to that more widely reported to fungi and plants^37^. We suggest that this gene transfer is the evolutionary result of a sustained endosymbiotic relationship between amoebozoa and bacteria, similar to that hypothesised for the acquisition of PALs in other kingdoms. This proposal aligns with reports of primitive agriculture between *Dictyostelium discoideum* and their bacterial livestock^47^, an interaction which could result in xenologous proteins in these otherwise unrelated organisms.

**Figure 4:**
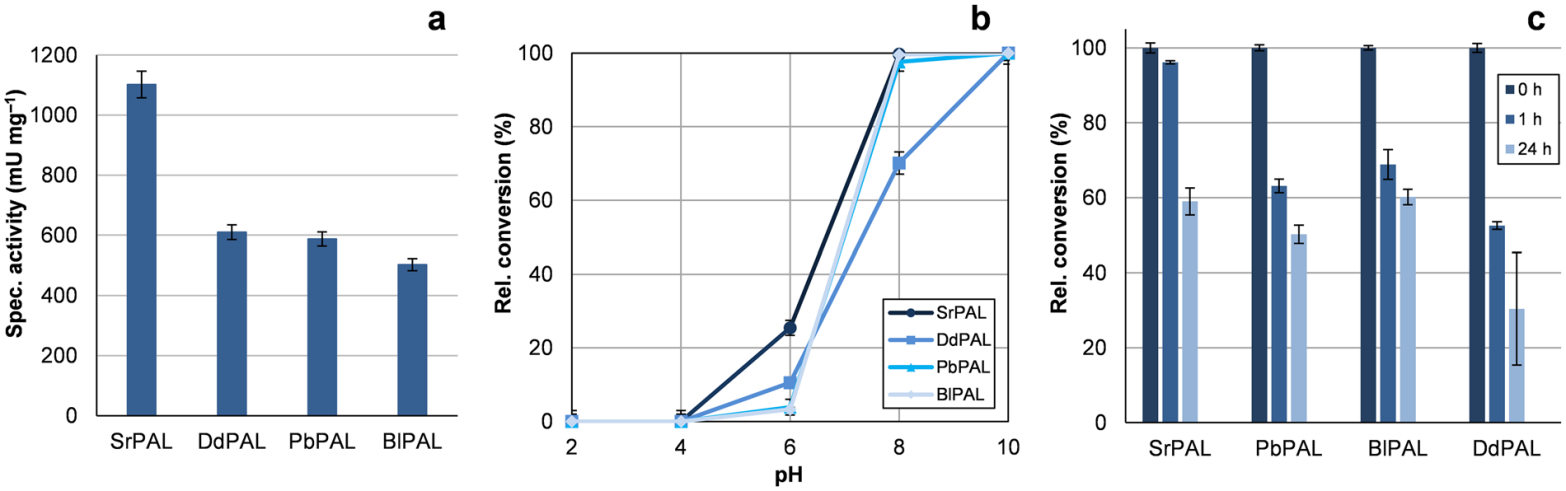
Variation of specific activity (**a**), pH tolerance (**b**) and stability at 37 °C (**c**) among four new ammonia lyases from *Streptomyces rimosus*, *Brevibacillus laterosporus*, *Planctomyces brasiliensis* and *Dictyostelium discoideum* which could be isolated for investigation.

Of the enzymes selected for further study, BlPAL and SrPAL were cloned from their host organisms whereas the genes for DdPAL and MxPAL were custom synthesised as codon-optimised constructs for expression in laboratory strain *E. coli*. These genes were then subcloned into expression plasmids. The gene encoding PbPAL was prepared and provided in an appropriate vector by collaborators at Prozomix Limited. After production of all five proteins as *N*-terminal His_6_-tagged variants, and successful purification of four, each enzyme was characterised with respect to amino acid specificity, pH optimum and stability at 37 °C (Fig. 4). To verify that these catalysts did not possess TAL or HAL activity, the isolated enzymes were incubated with L-Tyr or L-His and production of the corresponding deamination products assayed spectrophotometrically. In all cases, significant increases in absorbance, indicating acrylic acid production, were not seen with either L-Tyr or L-His (SI, Fig. S8). All four enzymes possessing exclusively PAL-activity were also shown to be misannotated in UniProtKB, with all designated as HALs except for BlPAL which was incorrectly assigned TAL function (Table 1). These incorrect designations, widespread in public databases^1^, impair the efforts of scientists in the search for new disease treatments, biocatalyst templates and components of unknown metabolic pathways. The case of enzymes with PAL activity illustrates this prominently, as correct annotation of these enzymes enables advances to be made in all three of these areas (e.g treatment of cancer/PKU, production of unnatural amino acids, identification of operons for bioactive molecules like basiliskamides).

To confirm PAL activity (rather than TAL or HAL) the specific activities with respect to the deamination of L-Phe were tested with each enzyme. Additionally, relative conversions after treatment under different conditions were also compared to assess the natural variation in therapeutically-relevant traits such as stability at blood and / or gastrointestinal temperature and pH (Fig. 4). The specific activity measurements made with each ammonia lyase indicated that SrPAL was the most active enzyme in the group, with a specific activity of 1102 mU mg^–1^, a value far higher than that of the other enzymes which all gave ~500 mU mg^–1^. The differences in specific activity between the enzymes were striking, particularly as SrPAL was shown to be far more active than the others with greater retention of activity after incubation at 37 °C. This difference was of particular interest as SrPAL and BlPAL share the closest sequence identity of any two enzymes in this study (SI, Fig. S6) and yet have a two-fold difference in turnover rate. These sequences, although closely related, possess many amino acid substitutions which are likely to influence the dynamics of the entire protein, possibly accelerating movements constituting the catalytic cycle or channelling solvent vibrations to the active site to help overcome activation energy^48,49^. The identical active site, yet variable overall sequence of SrPAL compared to BlPAL, seems to contribute to the former enzyme being a better candidate as a biotherapeutic, due to its high activity and longevity at average body temperature after 1 hour. However, after 24 hours all enzymes still allowed 50–60% of their initial maximal conversion with only DdPAL showing a greater loss in activity. SrPAL was also found to be the most acid tolerant of the enzymes along with DdPAL, which, whilst being less active and less robust initially, still retained more activity at pH 6 that either BlPAL or PbPAL. This feature may make SrPAL (or even DdPAL) a good starting point for the development of an acid tolerant enzyme for oral administration, ensuring function and stability in the digestive tract. These variable findings with no one protein possessing all the most desirable traits, highlight the utility of our simple method for estimating the viability of enzyme engineering by sampling variation capacity in a handful of diverse but related natural sequences.

In order to evaluate the synthetic potential of each enzyme, the *E. coli* cell pastes prepared for protein purification were used as whole cell biocatalysts for the amination of a range of ring-substituted arylacrylates. Following a reported method^30^, the biotransformations were run with all five enzymes and a representative panel of 16 cinnamate substrates **1a-p** before isolation of crude product and analysis (Table 2). Interestingly, despite the biocatalysts having nearly identical active sites, there was evident variability in substrate tolerance and enantioselectivity, aside from general trends expected from previous investigations (*e.g*., low enantioselectivity with **1n-p**)^36,50^. For instance, both SrPAL and PbPAL were found to give very high conversions for the majority of substrates but the enantioselectivity of the reaction catalysed by the former enzyme was superior in most cases. When compared to BlPAL however, SrPAL gave similarly excellent enantiomeric excess values across the board but much less variable conversion. A comparison between the three most discernible substrate profiles of BlPAL, MxPAL and DdPAL revealed that even the relative conversions were not the same between the enzymes. Whilst BlPAL gave the highest conversion for **1g**, followed by MxPAL and then DdPAL (76%, 56% and 28%) this pattern was reversed with the *ortho*-isomer 1e (12%, 63% and 84%) and different again for the *meta-*isomer **1f** (81%, 51% and 57%). Another interesting feature shared by MxPAL and DdPAL is that they both convert the natural ligand **1a** to a lower extent than most of its ring-substituted derivatives. DdPAL even gave higher conversion with the seemingly challenging substrate **1l**, a compound not even accepted by BlPAL or MxPAL and poorly converted by the otherwise highly active SrPAL.

**Table 2:** PAL-catalysed amination of various ring-substituted phenylacrylate derivatives.

1	R	SrPAL		BlPAL		PbPAL		MxPAL		DdPAL	
		Conv.^a^ (%)	ee^b^ (%)	Conv.^a^ (%)	ee^b^ (%)	Conv.^a^ (%)	ee^b^ (%)	Conv.^a^ (%)	ee^b^ (%)	Conv.^a^ (%)	ee^b^ (%)
**1a**	**H**	92	>99 (*S*)	80	>99 (*S*)	91	>99 (*S*)	11	—	25	95 (*S*)
**1b**	**2-F**	98	>99 (*S*)	99	>99 (*S*)	99	94 (*S*)	91	>99 (*S*)	60	88 (*S*)
**1c**	**3-F**	96	>99 (*S*)	96	>99 (*S*)	91	88 (*S*)	89	>99 (*S*)	58	82 (*S*)
**1d**	**4-F**	92	>99 (*S*)	92	>99 (*S*)	94	96 (*S*)	53	96 (*S*)	33	>99 (*S*)
**1e**	**2-Br**	99	96 (*S*)	12	>99 (*S*)	99	94 (*S*)	63	82 (*S*)	84	90 (*S*)
**1f**	**3-Br**	97	>99 (*S*)	81	>99 (*S*)	98	86 (*S*)	51	>99 (*S*)	57	82 (*S*)
**1g**	**4-Br**	96	>99 (*S*)	76	>99 (*S*)	97	96 (*S*)	56	>99 (*S*)	28	>99 (*S*)
**1h**	**2-Cl**	99	>99 (*S*)	6	>99 (*S*)	99	94 (*S*)	29	>99 (*S*)	85	86 (*S*)
**1i**	**3-Cl**	91	96 (*S*)	23	>99 (*S*)	97	91 (*S*)	34	>99 (*S*)	74	80 (*S*)
**1j**	**4-Cl**	82	>99 (*S*)	35	>99 (*S*)	70	92 (*S*)	22	>99 (*S*)	41	>99 (*S*)
**1k**	**2-MeO**	<1	—	<1	—	27	96 (*S*)	<1	—	15	>99 (*S*)
**1l**	**3-MeO**	19	85 (*S*)	<1	—	92	99 (*S*)	<1	—	40	96 (*S*)
**1m**	**4-MeO**	<1	—	<1	—	8	99 (*S*)	<1	—	<1	—
**1n**	**2-NO**_**2**_	97	87 (*S*)	39	88 (*S*)	99	70 (*S*)	19	60 (*S*)	70	58 (*S*)
**1o**	**3-NO**_**2**_	98	88 (*S*)	54	92 (*S*)	99	88 (*S*)	38	86 (*S*)	68	62 (*S*)
**1p**	**4-NO**_**2**_	92	70 (*S*)	37	88 (*S*)	99	32 (*S*)	36	44 (*S*)	67	78 (*S*)

^a^Determined by reverse phase HPLC on a non-chiral phase. ^b^Determined by reverse phase HPLC on a chiral phase.

**Figure 5:**
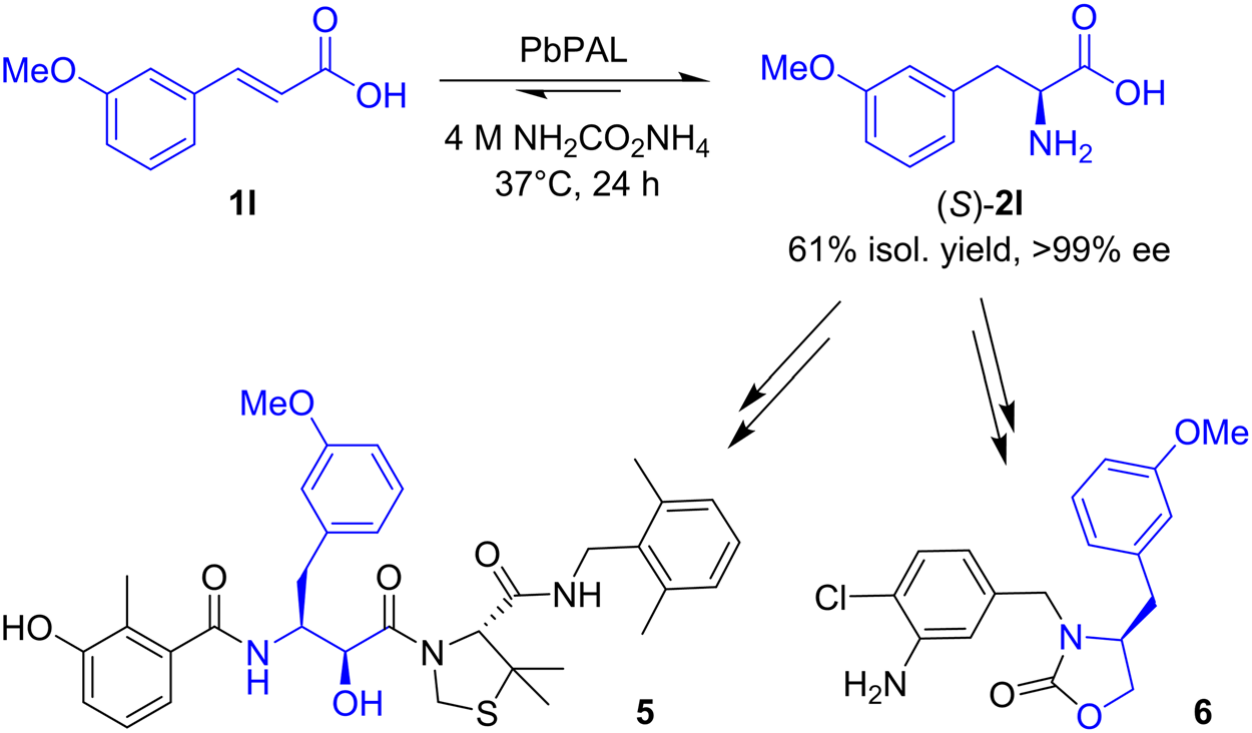
Preparative scale synthesis of L-*m*-methoxyphenylalanine (*S*)-**2l** by the newly discovered ammonia lyase from *Planctomyces brasiliensis* (PbPAL) and examples of antiviral and antidiabetic compounds **5** and **6** which can be synthesised from this building block.

The discovery that this new panel of enzymes showed high activity with substrates possessing electron-donating substituents was striking, as this class of substrate is not reported to be well accepted by PAL enzymes. Production of L-*m*-methoxyphenylalanine (*S*)-**2l** by three of the new PALs is of particular interest, as this unnatural amino acid is a relevant building block for pharmaceutical synthesis (Fig. 5). Examples of APIs that can be synthesised from this amino acid are antiviral peptides, such as **5** ^51^, and oxazolidinone antidiabetics, such as **6** ^52^. In light of this, a preparative scale amination of **1l** was performed using PbPAL, due to its superior enantioselectivity and impressive conversions compared to SrPAL and DdPAL. By repeating the biotransformation with a 10-fold increase in substrate loading and reaction volume (with 50 mg mL^−1^ whole cells) an isolated yield of 61% enantiomerically pure L-amino acid (ee > 99%) was obtained from 89 mg of starting material.

The differences in relative conversion and enantioselectivity of the biocatalysts (selected based on the similarity of their zymophore) also allude to the often overlooked contribution of non-active site residues to catalysis. These distal residues could coordinate breathing motions across the entire enzyme structure which may direct the malleability of the active site and modulate substrate accommodation. This has already been speculated within this class of enzymes, with the discovery that active site residues alone cannot discriminate closely related ammonia lyase and aminomutase enzymes in plants^53^. Although protein dynamics are known to influence enzyme activity^48,49^, there is little evidence of their consideration in biocatalyst or biotherapeutic research and development. Thus, this method of sampling sequence diversity outside the active site of an enzyme could be a crucial step in the investigation of these phenomena. The varying overall sequence-dynamics relationship of each enzyme may be the reason why, for example, PbPAL shows excellent activity with the novel substrate *m*-methoxycinnamate, whereas BlPAL, which has an identical active site, shows none. These underinvestigated considerations of protein dynamics for biocatalysis (and also enzyme replacement therapy) will hopefully benefit from dedicated investigation of these phenomena in future work.

Compounds with electron-donating ring-substituents have been considered difficult for PALs, presumably due to deactivation of the α-carbon as a result of the relative strengths of the aromatic ring and carboxyl group of these substrates as opposing electron sinks. This theory is thought to explain the conversely high activity of substrates (such as nitrocinnamates) with other ammonia lyases^36,50^. The discovery of PbPAL emphasises the difficulty with which such an activity would have been arrived upon using current engineering techniques without targeting active site residues, especially in the absence of a high-throughput enzyme assay. In this respect, our discovery method offers many advantages over traditional diversification across an entire parent sequence, which often involves lengthy and iterative mutagenesis yielding many poorly active variants, few useful hits and inherent problems with screening^6,54^. As such, we believe our method provides a complementary strategy to currently available techniques, particularly for selecting specific catalytic activities from functionally diverse families, such as other amino acid degrading enzymes, imine reductases/reductive aminases, esterases/hydrolases and nitrilases/nitrile hydratases.

## Methods

### X-ray crystallography

AvPAL-Y78F-C503S-C565S was purified and crystallized as described previously for other AvPAL variants^36^. Diffraction data was processed and the structure solved by molecular replacement of the AvPAL-C503S-C565S structure (PDB ID: 3CZO) as a search model. The crystal structure of AvPAL-Y78F-C503S-C565S bound to cinnamate was then deposited in the protein data bank (PDB ID: 5LTM).

### Sequence alignments

Potential PAL enzymes were identified from within the Universal Protein Resource KnowledgeBase (UniProtKB) with the basic local alignment search tool (BLAST) as provided by the UniProt website. All multiple amino acid sequence alignments were performed through use of the ClustalW2 programme using standard parameters as available online.

### Whole cell biocatalyst preparation

The codon-optimized open reading frames (ORFs) encoding MxPAL and DdPAL and wild-type sequences for BlPAL and SrPAL were subcloned into a standard pET-28a or pET-28b vector by use of relevant multiple cloning sites. The pET-28b-PbPAL construct was used as provided by Prozomix Ltd. from their initial ammonia lyase panel screening efforts. Transformed *E. coli* BL21 (DE3) strains were grown in 800 mL LB-based autoinduction medium supplemented with kanamycin (50 μg mL^−1^) at 18 °C (250 rpm agitation) for 4 days. Cells were harvested via centrifugation and stored at −20°C until further use.

### Whole cell biotransformations

The whole cell formulation was used directly as stored by resuspending in an appropriate volume of unadjusted 4 M ammonium carbamate (pH ~9.9) supplemented with the relevant arylacrylic acid **1a-p** at the required concentration (10 mM for screening, 50 mM for preparative applications). All biotransformations were performed at 37 °C with agitation of 250 rpm. After a 24 h incubation period, a sample was subjected to centrifugation (3 min, 13000 rpm) to remove the catalyst before evaporation of the volatile reaction medium. The crude isolate was then dissolved in an appropriate solvent for analysis by HPLC.

## Electronic supplementary material

Supplementary Information
