# Supplementary material for: Zymophore identification enables the discovery of novel phenylalanine ammonia lyase enzymes
Source: Sci Rep. 2017 Oct 20;7:13691. doi: 10.1038/s41598-017-13990-0 (PMC5651878; doi:10.1038/s41598-017-13990-0)
Supplement: Supplementary file 1 — Supplementary Information [file 41598_2017_13990_MOESM1_ESM.pdf]

SUPPLEMENTARY INFORMATION (SI)

Zymophore identification enables the discovery of novel phenylalanine ammonia lyase enzymes

Nicholas J. Weise[a], Syed T. Ahmed[a], Fabio Parmeggiani[a], James L. Galman[a], Mark S. Dunstan[b], Simon J. Charnock[c], David Leys[a,b], and Nicholas J. Turner[a,b]*

[a] *School of Chemistry,* [b] *SYNBIOCHEM, Manchester Institute of Biotechnology, University of Manchester, 131 Princess Street, Manchester, M1 7DN, United Kingdom*

[c] *Prozomix Ltd. Station Court, Haltwhistle, Northumberland, NE49 9HN, United Kingdom*

Supplementary materials and methods

General methods

Analytical grade reagents and solvents were obtained from Sigma-Aldrich, AlfaAesar or Fisher Scientific and used without further purification, unless stated otherwise. Reference standards of optically pure amino acids were purchased from PepTech Corporation.

Plasmid DNA purification was performed with the QIAprep Spin miniprep kit (Qiagen), according to the manufacturer's instructions. Automated DNA sequencing was performed by MWG Eurofins.

E. coli DH5α was used as a cloning host for plasmid propagation and *E. coli* BL21(DE3) as an expression host for protein production. Chemically competent cells of both strains were purchased from New England Biolabs. Solid and liquid media were supplemented with kanamycin (50 μg mL^{-1} final concentration). Solid media were prepared by addition of agar (1.5% w/v) to liquid media.

The pET-28a-PbPAL plasmid (pET-28a vector containing the codon-optimised gene encoding the PbPAL protein) was obtained from collaborators at Prozomix Ltd.

Molecular visualisations and modelling

The protein structure for the ammonia lyase AvPAL was downloaded from the Research Collaboratory for Structural Bioinformatics' Protein Data Bank – RCSB PDB – www.rcsb.org (PDB ID: 3CZO). This was used to form an overlaid visualisation of the occupied and empty AvPAL active sites with the YASARA molecular modelling software (version 14.7.17). Correct positioning of the two structures was achieved using the superpose function as applied to both structures as loaded objects.

X-ray crystallography

AvPAL-Y78F-C503S-C565S was purified as described below and concentrated in a centrifugal spin concentrator (Vivaspin 30 kDa cut-off) to a final concentration of 15 mg mL^{-1}. Crystallisation occurred in conditions similar to those reported for the AvPAL-C503S-C565S,[S1] with rod shaped crystals appearing in a broad range of PEG1500 concentrations (12-20%) and 100 mM SPG buffer (succinic acid, sodium dihydrogen phosphate, glycine, pH 7.0). Protein and reservoir solution were mixed in a 1:1 ratio and incubated at 25°C for 3 days. Crystals were cryoprotected in 25% glucose before being flash-frozen in liquid nitrogen. Data was collected at Diamond Light Source (Didcot, UK) on station i24, from a single cryofrozen crystal. Data was processed and scaled using XDS and the structure solved by molecular replacement using PHASER and the AvPAL-C503S-C565S structure (PDB ID: 3CZO) as a search model. Iterative cycles of rebuilding and refinement were carried out in COOT and Phenix Refine. The crystal structure of AvPAL-Y78F-C503S-C565S bound to cinnamate has been deposited in the protein data bank (PDB ID: 5LTM).

Database searches

Discovery of uncharacterised potential enzyme sequences was undertaken using an appropriate query (protein sequence of ammonia lyases from *Anabaena variabilis*, *Photorhabdus luminescens* and *Streptomyces* sp.) to perform a sequence similarity search within the knowledge base of the universal protein resource. In each case the basic local alignment search tool (BLAST) was used to find regions of sequence similarity between an amino acid sequence and the *in silico* translated DNA sequences of all available genomes and metagenomes (tBLASTn). All searches were gapped and unfiltered with a statistical significance value threshold of E=10. The choice of protein substitution matrix was set to automatic and thus assigned computationally based on the length of sequence. Sequences were downloaded in fasta format for inspection and alignment. All sequence alignments were performed through use of the W2 command line interface for the Clustal multiple sequence alignment computer programme, as available online. All alignments made use of the Gonnet protein weight matrix with the 'gap open' penalty score set to 10 in all cases. The initial pairwise alignment type was set to slow with a gap extension score of 0.1. With subsequent multiple alignments the gap extension score was set to 0.2 in addition to a gap distance penalisation value of 5, without end gap penalisation or iteration. Sequences were clustered via the neighbour-joining method. Alignments of all putative PALs were performed against the primary sequence of AvPAL to allow accurate mapping of the zymophore motif onto homologous positions.

Cloning of Wild-type BlPAL and SrPAL Sequences

The wild-type sequences for SrPAL and BlPAL were cloned and amplified by colony PCR from *Brevibacillus laterosporus* (NCIMB 701124) and *Streptomyces rimosus* subsp. *rimosus* (NCIMB 8229) using the following primers (restriction sites underlined):

SrPAL-Fw: 5'-ACCTGCATATGCACACCATGGACACTGCCCTGGCAGCCAACG-3'

SrPAL-Rv: 5'-TCGCACTCGAGTCAGTCCCGCCCGGCGTCCTCGACGGCCCGGCGGAGC-3'

BlPAL-Fw: 5'-GCTTACATATGAGTCAAGTAGCCCTTTTCGAACAAGAGTTGATGC-3'

BlPAL-Rv: 5'-TCGATGGATCCCTAGTCATTCACATTTTGATCGTGAATTTCTTTC-3'

The following touchdown PCR protocol was used in the first phase: 3 min initial denaturation at 98°C and then 15 cycles of 30 s denaturation at 98°C, 30 s annealing at 65°C (–1°C each cycle) and 2 min elongation at 72°C. The second phase involved: 20 cycles of 30 s denaturation at 98°C, 30 s annealing at 55°C, and 2 min elongation at 65°C, with 5 min final extension time at 72°C. After PCR product purification each amplicon was subcloned into pET-28b between the *Nde*I and *Xho*I restriction sites for SrPAL or *Nde*I and *Bam*HI sites for BlPAL.

HPLC analysis

Reverse phase HPLC analyses were performed on an Agilent 1200 Series system equipped with a G1379A degasser, G1312A binary pump, a G1329 autosampler unit, a G1316A temperature controlled column compartment and a G1315B diode array detector.

Conversion analysis was performed on a ZORBAX Extend-C18 column (50 mm × 4.6 mm × 3.5 µm Agilent). Mobile phase: NH_4OH buffer (0.35% w/v, pH 10.0) / MeOH (see Table S2). Flow rate: 1 mL min^{-1}. Temperature: 40°C. Detection wavelength: 210 nm. Peaks were assigned via comparison with commercially available standards. Conversions were calculated from peak area integrations with use of appropriate response factors where needed.

Enantiomeric excesses were measured using a CROWNPAK CR(+) HPLC column, (150 mm × 4 mm × 5 µm, Daicel). Mobile phase: aq. $HClO_4$ (1.14% w/v, pH 2.0) / MeOH (see Table S3). Flow rate: 1 mL min^{-1}. Temperature: 25°C. Detection wavelength: 210 nm. Peaks were assigned via comparison with the literature[S2] and with commercially available standards. Enantiomeric excess values were calculated from peak area integrations.

Retention times are given in Tables S2 and S3 for separation of authentic standards using methods based on previous investigations.[S3]

Purification of proteins

E. coli BL21(DE3) whole cells containing the overproduced enzyme (3.5 g wet weight) were resuspended in wash buffer (25 mL, 50 mM KPi, 500 mM NaCl, 20 mM imidazole, pH 7.4). Lysozyme (500 µL, 10 mg mL^{-1}) was added and the mixture was incubated at 37°C and 220 rpm for 45 min. The suspension was sonicated (20 s on, 20 s off, 20 cycles, Soniprep 150, MSE UK Ltd) on ice and treated with DNAse (100 µL, 1 mg mL^{-1}) at 37°C and 220 rpm for 45 min. The mixture was centrifuged (18,000 rpm, 30 min, 4°C) and the supernatant was filtered (0.2 µm syringe filter) and loaded onto a prepacked HisTrap FF column (GE Healthcare, 1 mL solid phase). The column was washed with the wash buffer (5-10 mL) and the protein was eluted with the elution buffer (10 mL, 50 mM KPi, 500 mM NaCl, 250 mM imidazole, pH 7.4), collecting the eluate in different fractions. Fractions were pooled according to the protein concentration (measured by Bradford assay) with sufficient purity (judged by SDS-PAGE analysis) and used without further processing.

Calculation of specific activities

The purified PAL (50 µL of a 0.5 mg mL^{-1} solution in NaP_i buffer pH 7.4) was added to a solution of L-phenylalanine (20 mM, 225 µL) and borate buffer (675 µL, 100 mM, pH 10.0). The mixture was incubated at 37°C, 220 rpm for 1 h. Samples were analysed by HPLC on a non-chiral stationary phase. For the calculation, 1 unit is defined as the amount of enzymes converting 1 µmol of L-**2a** to **1a** in 1 min.

Purified enzyme plate reader assay

A solution containing 0.5 mg mL^{-1} enzyme (20 µL) was added to a 96-well plate followed by addition of substrate solution (L-Tyr or L-His, 180 µL, 5 mM) to a total volume of 200 µL. The assay was performed at 37°C for 20 minutes measuring at 30 s intervals. Detection wavelengths: coumaric acid 380 nm, urocanic acid 320 nm.

Purified enzyme analytical scale assay

A solution containing 0.5 mg mL^{-1} enzyme (50 µL) was added to solution of either borate buffer (430 µL, 100 mM, pH 8.0-10.0) or NaP_i (430 µL 100 mM, pH 6.0), followed by addition of L-phenylalanine solution (20 µL, 250 mM in the same buffer) to a final substrate concentration of 5 mM and a final enzyme concentration of 0.05 mg mL^{-1}. Temperature stability tests were conducted by incubating the enzyme in borate buffer pH 8.0 for the specified time (1, 24 and 48 h) at 37°C, followed by addition of L-phenylalanine (20 µL 250 mM) and incubation for a further 16 h. Results for these were reported as conversions relative to the maximum obtained with the untreated cells.

Workup for the preparative scale synthesis of amino acid 2l

Supernatant from the reaction mixture was acidified to pH < 2 by addition of aqueous H_2SO_4 (10% w/v) and centrifuged (4000 rpm, 10 min, 4°C) to remove cells and insoluble components. Dowex 50WX8 hydrogen form (2.5 g) was washed with deionised water (50 mL) and aqueous H_2SO_4 (25 mL, 10% w/v). The acidified supernatant from the biotransformation was loaded onto the resin (1 mL min^{-1}). The resin was washed repeatedly with deionised water (until pH ~7.0) and the product was eluted with aqueous NH_4OH (30 mL, 10% w/v). Fractions containing the product were pooled and evaporated in a centrifugal evaporator, to afford amino acid L-**2l**.

Characterisation data of (*S*)-2l from preparative scale biotransformation

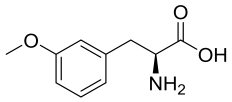

(*S*)-2-amino-3-(3-methoxyphenyl)propanoic acid. White crystals, 61% yield; ^{1}H NMR (400 MHz, D_2O+NaOH): 7.24 (t, *J* = 8 Hz, 1H), 6.81-6.84 (m, 3H), 3.76 (m, 3H), 3.43 (t, *J* = 8, 1H), 2.88-2.93 (dd, *J* = 16, 8 Hz, 1H), 2.73-2.78 (dd, J = 12, 8 Hz, 1H); ^{13}C NMR (101 MHz, D_2O+NaOH): 182.30, 158.79, 140.11, 129.70, 122.27, 114.83, 112.22, 57.32, 55.26, 40.74; HRMS (*m/z*): $[M]^+$ calcd. for $C_{10}H_{14}NO_3$,196.0895; found 196.1017.

Supplementary figures

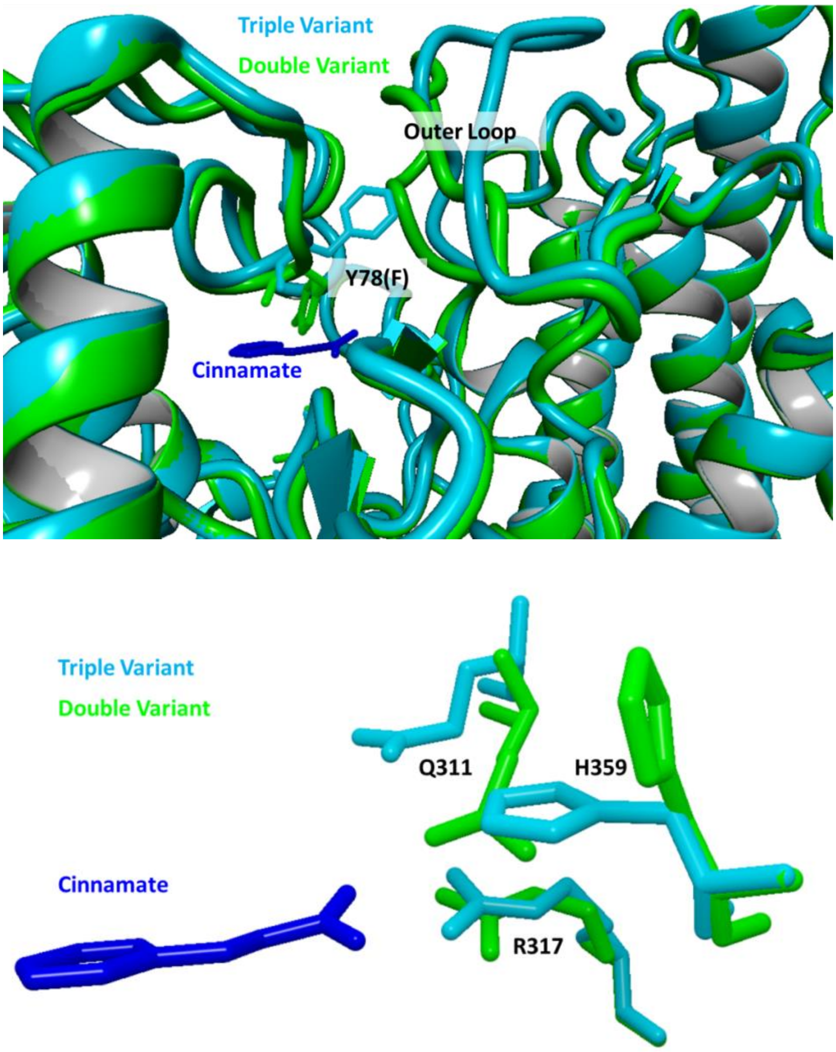

Figure S1. Overlaid structures of the AvPAL triple variant structure with bound cinnamate (solved in this study, PDB ID: 5LTM) and the ligand-free double variant structure (reported previously, PDB ID: 3CZO).

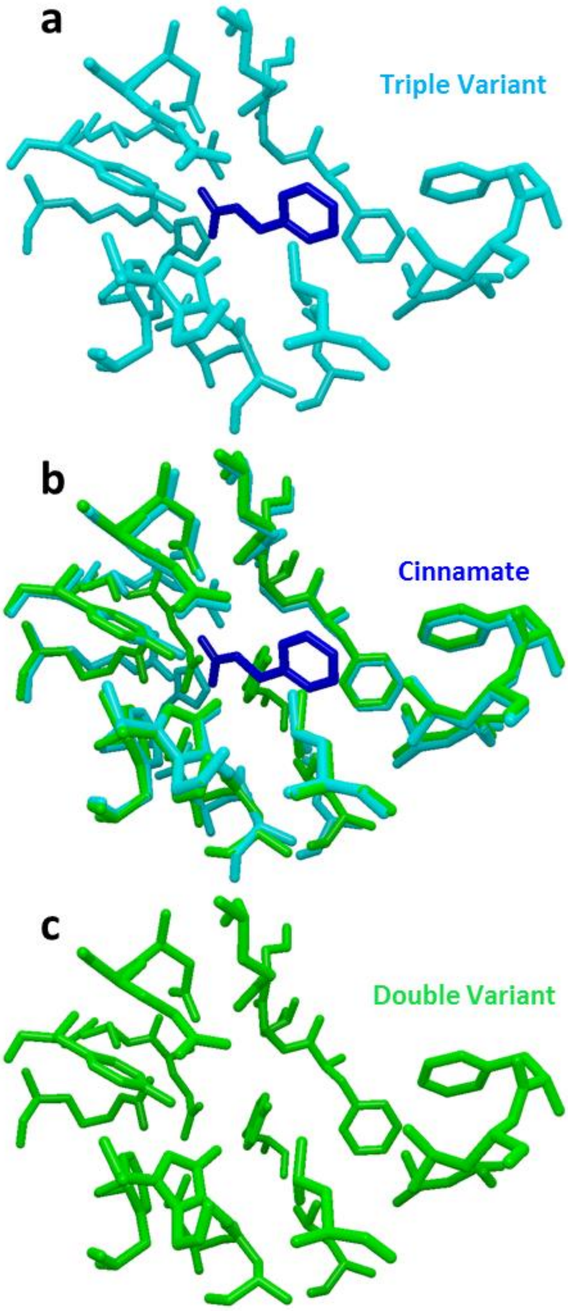

Figure S2. (a) the zymophore conferring PAL-specific chemistry and selectivity as identified from the co-crystal structure of the ammonia lyase from *Anabaena variabilis* (AvPAL) and its deamination product *trans*-cinnamate (PDB ID: 5LTM). (b) an overlay of the existing active site structure of AvPAL with the co-crystal structure from this work. (c) the previously reported empty active site structure of AvPAL (PDB ID: 3CZO).

```
...

SgTAM    2(2)   LTVEAVRRVA-EERAT---VDVPAESIAKAQKSREIFEGIAEQNIPIYGVTTGYGEMIYM 75
CmdF     1(1)   LSIYDVADVC-MKRAT---VELDPSQLERVAVAHERTQAWGEAQHPIYGVNTGFGELVPV 63
PpHAL    1(1)   LTLAQLRAIH-AAPVR---LQLDASAAPAIDASVACVEQIIAEDRTAYGINTGFGLLAST 66
AdmH     2(2)   ISLEEIARAA-RDHQP---VTLHDEVVNRVTRSRSILESMVSDERVIYGVNTSMGGFVNY 90
BagA     1(1)   LTISQTVAAASREGSE---FAVSEDALRAMNASRNLKLEILATGKPIYGVTTGFGDSVNR 64
StlA     0(0)   ISLEDIYDIA-IKQKK---VEISTEITELLTHGREKLEEKLNSGEVIYGINTGFGGNANL 73
AvPAL    -(-)   LTINDVARVARNGTL--VSLTNNTDILQGIQASCDYINNAVESGEPIYGVTSGFGGMANV 90
TcPAM    1(1)   ITVAHVAALARRHDVK--VALEAEQCRARVETCSSWVQRKAEDGADIYGVTTGFGACSSR 92
PcPAL1   1(1)   LTISQVAAISARDGSG-VTVELSEAARAGVKASSDWVMDSMNKGTDSYGVTTGFGATSHR 122
RtPAL    0(0)   LNLGDVVSAARKGRP--VRVKDSDEIRSKIDKSVEFLRSQLSM--SVYGVTTGFGGSADT 122
Sam8     1(1)   SSREYLARVVRSAGWDAGLTSCTDEEIVRMGASARTIEEYLKSDKPIYGLTQGFGP--LV 70

SgTAM    2(4)   QVDKSKEVELQTNLVRSHSAGV--------------GPLFAEDEARAIVAARLNTLAKGH 121
CmdF     2(3)   MIPRQHKRELQENLIRSHAAGG--------------GEPFADDVVRAIMLARLNCLMKGY 109
PpHAL    2(3)   RIASHDLENLQRSLVLSHAAGI--------------GAPLDDDLVRLIMVLKINSLSRGF 112
AdmH     2(4)   IVPIAKASELQNNLINAVATNV--------------GKYFDDTTVRATMLARIVSLSRGN 136
BagA     2(3)   QISPEKTARLQNELIRYHLNGT--------------GQLASDEVVRATVLIRANCLARGN 110
StlA     0(0)   VVPFEKIAEHQQNLLTFLSAGT--------------GDYMSKPCIKASQFTMLLSVCKGW 119
AvPAL    -(-)   AISREQASELQTNLVWFLKT--------------GAGNKLPLADVRAAMLLRANSHMRGA 136
TcPAM    1(2)   RTN--RLSELQESLIRCLLAGVFTKG------CAPSVDELPATATRSAMLLRLNSFTYGC 144
PcPAL1   1(1)   RTK--QGGALQKELIRFLNAGIFGNG-------SD--NTLPHSATRAAMLVRINTLLQGY 171
RtPAL    2(2)   RTE--DAISLQKALLEHQLCGVLPSSFDSFRLGRGLENSLPLEVVRGAMTIRVNSLTRGH 180
Sam8     1(2)   LFDADSELEQGGSLISHLGTGQ--------------GAPLAPEVSRLILWLRIQNMRKGY 116

SgTAM    0(4)   SAVRPIILERLAQYLNEGITPAIPEIGSLGASGDLAPLSHVASTLIGEG-----YVLR-D 175
CmdF     0(3)   SGASVETVKLLAEFINRGIHPVIPQQGSLGASGDLSPLSHIALALIGEG-----TVSF-K 163
PpHAL    0(3)   SGIRRKVIDALIALVNAEVYPHIPLKGSVGASGDLAPLAHMSLVLLGEG-----KARYKG 167
AdmH     1(5)   SAISIVNFKKLIEIYNQGIVPCIPEKGSLGTSGDLGPLAAIALVCTGQW-----KARY-Q 190
BagA     0(3)   SGVSLPVVELLLDFLKHDILPTVPERGSVGASGDLVPLCYLAYALTGQG-----KVRH-R 164
StlA     0(0)   SATRPIVAQAIVDHINHDIVPLVPRYGSVGASGDLIPLSYIARALCGIG-----KVYY-M 173
AvPAL    -(-)   SGIRLELIKRMEIFLNAGVTPYVYEFGSIGASGDLVPLSYITGSLIGLDPSFKVDFNG-- 194
TcPAM    0(2)   SGIRWEVMEALEKLLNSNVSPKVPLRGSVSASGDLIPLAYIAGLLIGKPSVIARIGDD-- 202
PcPAL1   0(1)   SGIRFEILEAITKFLNQNITPCLPLRGTITASGDLVPLSYIAGLLTGRPNSKAVGPTG-- 229
RtPAL    0(2)   SAVRLVVLEALTNFLNHGITPIVPLRGTISASGDLSPLSYIAAAISGHPDSKVHVVHEGK 240
Sam8     0(2)   SAVSPVFWQKLADLWNKGFTPAIPRHGTVSASGDLQPLAHAALAFTGVGEAWTRDADG-R 175

SgTAM    0(4)   GRPVETAQVLAERGIEP--LELRFKEGLALINGTSGMTGLGSLVVGRALEQAQQAEIVTA 233
CmdF     0(3)   GQVRKTGDVLREEGLKP--LELGFKGGLTLINGTSAMTGAACVALGRAYHLFRLALLATA 221
PpHAL    0(3)   -QWLPATEALAIAGLEP--LTLAAKEGLALLNGTQASTAYALRGLFQAEDLYAAAIACGG 224
AdmH     0(5)   GEQMSGAMALEKAGISP--MELSFKEGLALINGTSAMVGLGVLLYDEVKRLFDTYLTVTS 248
BagA     0(3)   GETRPTAEVLAELGLQP--VTLEAKDGLALINGTSFSAAFAVLNTEAAAELADVADICTA 222
StlA     0(0)   GAEIDAAEAIKRAGLTP--LSLKAKEGLALINGTRVMSGISAITVIKLEKLFKASISAIA 231
AvPAL    -(-)   -KEMDAPTALRQLNLSP--LTLLPKEGLAMMNGTSVMTGIAANCVYDTQILTAIAMGVHA 251
TcPAM    0(2)   -VEVPAPEALSRVGLRP--FKLQAKEGLALVNGTSFATAVASTVMYDANVLLLLVETLCG 259
PcPAL1   0(1)   -VILSPEEAFKLAGVEGGFFELQPKEGLALVNGTAVGSGMASMVLFEANILAVLAEVMSA 288
RtPAL    0(2)   EKILYAREAMALFNLEP--VVLGPKEGLGLVNGTAVSASMATLALHDAHMLSLLSQSLTA 298
Sam8     0(2)   WSTVPAVDALAALGAEP--FDWPVREALAFVNGTGASLAVAVLNHRSALRLVRACAVLSA 233

...

SgTAM    1(5)   GKDVQRSEIYLQKAYSLRAIPQVVGAVRDTLYHARHKLRIEL-NSANDNPLFFEG--KEI 350
CmdF     0(3)   GNDVVDTGVYLQDAYTLRAVPQILGPVLDTLDFARKLIEEEL-NSTNDNPLIFDVP-EQT 338
PpHAL    0(3)   ----------VQDPYSLRCQPQVMGACLTQLRQAAEVLGIEA-NAVSDNPLVFAAE-GDV 324
AdmH     1(6)   LVKAS--NHQIEDAYSIRCTPQILGPVADTLKNIKQTLTNEL-NSSNDNPLIDQTT-EEV 363
BagA     0(3)   -RHYQRLTRSIQDRYSLRCAPHVNGVLRDMLDWVRTWMTVEI-NSSSDNPLFDPST-GAV 337
StlA     1(1)   HQEITQLNDTLQEVYSIRCAPQVLGIVPESLATARKILEREV-ISANDNPLIDPEN-GDV 347
AvPAL    -(-)   ---------ELIQDRYSLRCLPQYLGPIVDGISQIAKQIEIEI-NSVTDNPLIDVDN-QAS 357
TcPAM    0(2)   ---------KPKQDRYALRSSPQWLAPLVQTIRDATTTVETEV-NSANDNPIIDHAN-DRA 365
PcPAL1   0(1)   ---------KPKQDRYALRTSPQWLGPQIEVIRSSTKMIEREI-NSVNDNPLIDVSR-NKA 394
RtPAL    0(2)   ---------ILRQDRYPLRTSPQWLGPLVSDLIHAHAVLTIEAGQSTTDNPLIDVEN-KTS 407
Sam8     1(3)   ----------LQEPYSLRCAPQVLGAVLDQLDGAGDVLAREV-DGCQDNPITYEG---EL 327

...
```

```
SgTAM    1(6)   GLHSGFAGAQYPATALVAENRTIG-PASTQSVPSNGDN-QDVVSMGLISARNARRVLSNN 462
CmdF     1(4)   GLLCGFEGGQYLATSIASENLDLAAPSSIKSLPSNGSN-QDVVSMGTTSARKSLRLCENV 451
PpHAL    2(5)   GVNSGFMIAQVTAAALASENKALSHPHSVDSLPTSANQ-EDHVSMAPAAGKRLWEMAENT 435
AdmH     1(7)   GLRLGLMGGQFMTASITAESRASCMPMSIQSLSTTGDF-QDIVSFGLVAARRVREQLKNL 476
BagA     1(4)   GLQHGFKGMQIACSSLTAEALKHSGPASTFSRSTEAHN-QDKVSMAPIAARDARTVIELT 456
StlA     0(1)   GMYQGFKGVQLSQTALVAAIRHDCAASGIHTLATEEYN-QDIVSLGLHAAQDVLEMEQKL 459
AvPAL    -(-)   KVNMGLKGLQICGNSIMPLLTFYGNSIADRFPTHAEQFNQNINSQGYTSATLARRSVDIF 472
TcPAM    0(2)   SVDYGLKGLDIAMAAYSSELQYLANPVTT-HVHSAEQHNQDINSLALISARKTEEALDIL 479
PcPAL1   0(1)   SLDYGFKGAEIAMASYCSELQFLANPVTN-HVQSAEQHNQDVNSLGLISSRKTSEAVEIL 508
RtPAL    0(2)   SLSYHCKGLDIAAAAYTSELGHLANPVTT-HVQPAEMANQAVNSLALISARRTTESNDVL 520
Sam8     1(4)   GRGAGLAGVQISATSFVSRIRQLVFPASLTTLPTNGWN-QDHVPMALNGANSVFEALELG 439
```

...

Figure S3. Sections of a sequence alignment of characterised class I lyase-like enzymes linking their known catalytic activity to the number of conserved / varied amino acids within the active site. Yellow residues show conservation at these positions whereas blue residues indicate variation and the number of variations in each line is given on the left hand side (cumulative number in brackets). The enzymes in the alignment are: the (*S*)-selective TAM from *Streptomyces globisporus* (SgTAM),[S4] the (*R*)-selective TAM from from *Chondromyces crocatus* (CmdF),[S5] the bacterial HAL from *Pseudomonas putida* (PpHAL),[S6] the (*S*)-selective PAM from *Pantoea agglomerans* (AdmH),[S7] two bacterial PALs from *Photorhabdus luminescens* (StlA)[S8] and *Anabaena variabilis* (AvPAL),[S1] the (*R*)-selective PAM from from *Taxus canadensis* (TcPAM),[S9] one of the PAL paralogues from *Petroselinum crispum* (PcPAL1),[S10] the bifunctional PAL/TAL from *Rhodosporidium toruloides* (RtPAL)[S11] and two distinct TALs from from *Streptomyces* sp. (BagA)[S12] and from *Saccharothrix espanaensis* (Sam8).[S13]

>DdPAL (Dictyostelium discoideum)

```
MIETNHKDNFLIDGENKNLEINDIISISKGEKNIIFTNELLEFLQKGRDQLENKLKENVA
IYGINTGFGGNGDLIIPFDKLDYHQSNLLDFLTCGTGDFFNDQYVRGIQFIIIIALSRGW
SGVRPMVIQTLAKHLNKGIIPQVPMHGSVGASGDLVPLSYIANVLCGKGMVKYNEKLMNA
SDALKITSIEPLVLKSKEGLALVNGTRVMSSVSCISINKFETIFKAAIGSIALAVEGLLA
SKDHYDMRIHNLKNHPGQILIAQILNKYFNTSDNNTKSSNITFNQSENVQKLDKSVQEVY
SLRCAPQILGIISENISNAKIVIKREILSVNDNPLIDPYYGDVLSGGNFMGNHIARIMDG
IKLDISLVANHLHSLVALMMHSEFSKGLPNSLSPNPGIYQGYKGMQISQTSLVVWLRQEA
APACIHSLTTEQFNQDIVSLGLHSANGAASMLIKLCDIVSMTLIIAFQAISLRMKSIENF
KLPNKVQKLYSSIIKIIPILENDRRTDIDVREITNAILQDKLDFINLNL
```

>MxPAL (Methylobacterium sp.)

```
MNSNQQIIVSGSRLSVDQIVEVGLHKRNLFLTCDPQLRRTINDAADFVYRAVANEEVVYG
INTNFGGMANQVLSLNEVEDLQQNLIWGLKCGVGKKLPAAQVRSAMFIRANMLAKGVSGA
RAELIERYLVFLNAGITPVVRDLGSIGASGDLVPLAQIAGCLIGLGPSFRVERDGEEMDA
LSALSMLNLQPLKLRAKEGLALVNGSSMMSSIGAHCVHDTRHLTRLALHVHAMLIQALNA
SSESFDPFIHQNKPHPGQIAVAAAMRHLLRGSKSLKPNGHRKADGSGSLLQDRYSVRCLP
QYLGPIVDGLHAIEGQIEVEANSVDDNPLIDLENERLLHGGNFFAEYVALGMDQLRTYMA
LLAKHLDVQIAFAVAPEFNSGLPASLVGDQDNRIKFGLKGLQICANSIVPKLLHLSNGIS
VLFPTHAEQFNQNINSQGFNSATLASESVSLFKQYLAISLVFGIQAMDLRARATGGGFDG
RRYLSPTLLPLYETVRALLGRPASDERPLVFRNDEQDLSDHVAAIVADLSRPGGEIIGAM
AAEFAPGAGPAFGSPATRAGGRVAVAP
```

>PbPAL (Planctomyces brasiliensis)

```
MLASSPSGHTNPVLSGAPLSINVVADIGRQRLIPSLTDDEQVLNRVHACRDVVQKAVRNN
ERIYGITTGFGGMSDIPIPPQHVAQTQDNLLAFLSTSTGASLDPRHVRAAMALRANVLLQ
GRSGVRLELIERLVEFLRQDAIPVVCDLGSIGASGDLVPLGVIARSIIGHPSTTQVKYQG
EQADSHDVLQQLNYSALQLEAKEGLALVNGTSFSSAIAANCVFESQRLLSLSLVLQSIMV
RALGGHPEAFHPFVDENKPHPGQGWSAQMMRDLLSYSPNDSKRNGDLAQDRYSLRCLAQY
FAPIVEGIAQISQSISTEMNAVSDNPLIDVDTGRFHQSGNFLGQYVAMSMDQLRRHLGLL
AKHLDVQIAQLVAPAFNNGLPASLRGNSSRPFNMGLKGLQITGNSIMPLLTYLGNPLTEH
FPTHAEEFNQNINGLSWGSANLAWRSVQLFQHYLSVASIFAVQAIDLRAGLEADHCDGRE
LLGETATELYETVYDLLERNCGQESPFLFNDDEQSLEVDLQMLNGDLAGAGRMHEAVSSV
TDSFLAEFCE
```

>SrPAL (Streptomyces rimosus)

```
MHTMDTALAANDKAELLIDGHTLTVADVVSGARPADTTRVRARLAEGAVQRIEQSLALKN
KVIEAGLPVYGVTSGFGDSNTRQISGLKSEALQTNLIRFLSCGIGPVATPDVIRATMIVR
ANCLARGASGIRTEILELLLDCLNNDVLPPIPERGSVGASGDLVPLSYVAALLTGQGKAL
HQGEEKDASAALADAGLGAVVLGAKEGLALVNGTSFMSGFATLAVHDATELAFAADLSTA
LASQVLQGNPGHFVPFIFDQKPHTGTRTSARTIRELLGNPEDCDPSVDPEGAALTESGFR
QLEEPIQDRYSVRCAPHVTGVLRDTLDWAKNWVEVEINSTNDNPLFDVEAGMVRNGGNFY
GGHVGQAMDALKTAVASVGDLLDRQLELIVDEKFNNGLTPNLIPRFDADSWEAGLHHGFK
GMQIAASGLTAEALKNTMPATSFSRSTEAHNQDKVSMATIAARDARTVVELVRQVAAIHL
LALCQAADLRGQECLSAPTRAAYELIRSVSATMDGDRPLARDIELVVGLIASGELRRAVE
DAGRD
```

>BlPAL (Brevibacillus laterosporus)

```
MSQVALFEQELMLHGKHTLLLNGNDLTITDVAQMAKGTFEAFTFHISEEANKRIEECNEL
KHEIMNQHNPIYGVTTGFGDSVHRQISGEKAWDLQRNLIRFLSCGVGPVADEAVARATML
IRTNCLVKGNSAVRLEVIHQLIAYMERGITPIIPERGSVGASGDLVPLSYLASILVGEGK
VLYKGEEREVAEALGAEGLEPLTLEAKEGLALVNGTSFMSAFACLAYADAEEIAFIADIC
TAMASEALLGNRGHFYSFIHEQKPHLGQMASAKNIYTLLEGSQLSKEYSQIVGNNEKLDS
KAYLELTQSIQDRYSIRCAPHVTGVLYDTLDWVKKWLEVEINSTNDNPIFDVETRDVYNG
GNFYGGHVVQAMDSLKVAVANIADLLDRQLQLVVDEKFNKDLTPNLIPRFNNDNYEIGLH
HGFKGMQIASSALTAEALKMSGPVSVFSRSTEAHNQDKVSMGTISSRDARTIVELTQHVA
AIHLIALCQALDLRDSKKMSPQTTKIYNMIRKQVPFVERDRALDGDIEKVVQLIRSGNLK
KEIHDQNVND
```

Figure S4. The full amino acid sequences of the 5 new PALs selected for characterisation in Fasta format.

```
SrPAL           ---MHTMDTALAANDKAELLIDG--HTLTVADVVSGARPADTTRVRARLAEGAVQRIEQS 55
BlPAL           MSQVALFEQELMLHGKHTLLLNG--NDLTITDVAQMAK-GTFEAFTFHISEEANKRIEEC 57
DdPAL           ---------MIETNHKDNFLIDGENKNLEINDIISISK----GEKNIIFTNELLEFLQKG 47
MxPAL           ------------MNSNQQIIVSG--SRLSVDQIVEVGL--HKRNLFLTCDPQLRRTINDA 44
PbPAL           -------MLASSPSGHTNPVLSG--APLSINVVADIGR--QRLIPSLTDDEQVLNRVHAC 49
                               :   ::.*    * :  : . .                 . :.

SrPAL           LALKNKVIEAGLPVYGVTSGFGDSNTRQISGLKSEALQTNLIRFLSCGIGPVATPDVIRA 115
BlPAL           NELKHEIMNQHNPIYGVTTGFGDSVHRQISGEKAWDLQRNLIRFLSCGVGPVADEAVARA 117
DdPAL           RDQLENKLKENVAIYGINTGFGGNGDLIIPFDKLDYHQSNLLDFLTCGTGDFFNDQYVRG 107
MxPAL           ADFVYRAVANEEVVYGINTNFGGMANQVLSLNEVEDLQQNLIWGLKCGVGKKLPAAQVRS 104
PbPAL           RDVVQKAVRNNERIYGITTGFGGMSDIPIPPQHVAQTQDNLLAFLSTSTGASLDPRHVRA 109
                     . :     :**:.:.**.     :.  .    * **:  *. . *        *.

SrPAL           TMIVRANCLARGASGIRTEILELLLDCLNNDVLPPIPERGSVGASGDLVPLSYVAALLTG 175
BlPAL           TMLIRTNCLVKGNSAVRLEVIHQLIAYMERGITPIIPERGSVGASGDLVPLSYLASILVG 177
DdPAL           IQFIIIIALSRGWSGVRPMVIQTLAKHLNKGIIPQVPMHGSVGASGDLVPLSYIANVLCG 167
MxPAL           AMFIRANMLAKGVSGARAELIERYLVFLNAGITPVVRDLGSIGASGDLVPLAQIAGCLIG 164
PbPAL           AMALRANVLLQGRSGVRLELIERLVEFLRQDAIPVVCDLGSIGASGDLVPLGVIARSIIG 169
                   :    * :* *. *  ::.     :. .  * :   **:*********. :*  : *

SrPAL           QG---KALHQGEEKDASAALADAGLGAVVLGAKEGLALVNGTSFMSGFATLAVHDATELA 232
BlPAL           EG---KVLYKGEEREVAEALGAEGLEPLTLEAKEGLALVNGTSFMSAFACLAYADAEEIA 234
DdPAL           KG---MVKYNEKLMNASDALKITSIEPLVLKSKEGLALVNGTRVMSSVSCISINKFETIF 224
MxPAL           LGPSFRVERDGEEMDALSALSMLNLQPLKLRAKEGLALVNGSSMMSSIGAHCVHDTRHLT 224
PbPAL           HPSTTQVKYQGEQADSHDVLQQLNYSALQLEAKEGLALVNGTSFSSAIAANCVFESQRLL 229
                      .  . :  :   .*   .  .: * :*********: . *...  .  .   :

SrPAL           FAADLSTALASQVLQGNPGHFVPFIFDQKPHTGTRTSARTIRELLGNPEDCDPSVDPEG- 291
BlPAL           FIADICTAMASEALLGNRGHFYSFIHEQKPHLGQMASAKNIYTLLEGSQLSKEYSQIVGN 294
DdPAL           KAAIGSIALAVEGLLASKDHYDMRIHNLKNHPGQILIAQILNKYFNTSDNNTKSSNITFN 284
MxPAL           RLALHVHAMLIQALNASSESFDPFIHQNKPHPGQIAVAAAMRHLLRGSKSLKPNGHRKAD 284
PbPAL           SLSLVLQSIMVRALGGHPEAFHPFVDENKPHPGQGWSAQMMRDLLSYS----PNDSKRN- 284
                  :    ::  . * .    :   : : * * *    *  :   :  .

SrPAL           -AALTESGFRQLEEPIQDRYSVRCAPHVTGVLRDTLDWAKNWVEVEINSTNDNPLFDVEA 350
BlPAL           NEKLDSKAYLELTQSIQDRYSIRCAPHVTGVLYDTLDWVKKWLEVEINSTNDNPIFDVET 354
DdPAL           ----QSENVQKLDKSVQEVYSLRCAPQILGIISENISNAKIVIKREILSVNDNPLIDPYY 340
MxPAL           ----------GSGSLLQDRYSVRCLPQYLGPIVDGLHAIEGQIEVEANSVDDNPLIDLEN 334
PbPAL           ------------GDLAQDRYSLRCLAQYFAPIVEGIAQISQSISTEMNAVSDNPLIDVDT 332
                             .  *: **:** .:  . : : :   .  :. *  :..***::*

SrPAL           GMVRNGGNFYGGHVGQAMDALKTAVASVGDLLDRQLELIVDEKFNNGLTPNLIPRFDADS 410
BlPAL           RDVYNGGNFYGGHVVQAMDSLKVAVANIADLLDRQLQLVVDEKFNKDLTPNLIPRFNNDN 414
DdPAL           GDVLSGGNFMGNHIARIMDGIKLDISLVANHLHSLVALMMHSEFSKGLPNSLSP------ 394
MxPAL           ERLLHGGNFFAEYVALGMDQLRTYMALLAKHLDVQIAFAVAPEFNSGLPASLVG-----D 389
PbPAL           GRFHQSGNFLGQYVAMSMDQLRRHLGLLAKHLDVQIAQLVAPAFNNGLPASLRG-----N 387
                  .  .*** . ::   ** ::  :. :.. *.  :   :   *...*. .*

SrPAL           WEAGLHHGFKGMQIAASGLTAEALKNTMPATSFS-RSTEAHNQDKVSMATIAARDARTVV 469
BlPAL           YEIGLHHGFKGMQIASSALTAEALKMSGPVSVFS-RSTEAHNQDKVSMGTISSRDARTIV 473
DdPAL           -NPGIYQGYKGMQISQTSLVVWLRQEAAPACIHS-LTTEQFNQDIVSLGLHSANGAASML 452
MxPAL           QDNRIKFGLKGLQICANSIVPKLLHLSNGISVLFPTHAEQFNQNINSQGFNSATLASESV 449
PbPAL           SSRPFNMGLKGLQITGNSIMPLLTYLGNPLTEHFPTHAEEFNQNINGLSWGSANLAWRSV 447
                 .  :  * **:**  ..:                  :* .**:  . .  ::  *   :

SrPAL           ELVRQVAAIHLLALCQAADLRGQ---------ECLSAPTRAAYELIRSVSA--------- 511
BlPAL           ELTQHVAAIHLIALCQALDLRDS---------KKMSPQTTKIYNMIRKQVP--------- 515
DdPAL           IKLCDIVSMTLIIAFQAISLRMKSIE-----NFKLPNKVQKLYSSIIKIIP--------- 498
MxPAL           SLFKQYLAISLVFGIQAMDLRARATGGGFDGRRYLSPTLLPLYETVRALLGRPASDERPL 509
PbPAL           QLFQHYLSVASIFAVQAIDLRAGLEADHCDGRELLGETATELYETVYDLLERNCGQESPF 507
                    .  ::  :   ** .**             :       *. :

SrPAL           -TMDGDRPLARDIELVVGLIASGE-------LRRAVEDAGRD---------------- 545
BlPAL           -FVERDRALDGDIEKVVQLIRSGN-------LKKEIHDQNVND--------------- 550
DdPAL           -ILENDRRTDIDVREITNAILQDK-------LDFINLNL------------------- 529
MxPAL           VFRNDEQDLSDHVAAIVADLSRPGGEIIGAMAAEFAPGAGPAFGSPATRAGGRVAVAP 567
PbPAL           LFNDDEQSLEVDLQMLNGDLAGAG------RMHEAVSSVTDSFLAEFCE--------- 550
```

Figure S5. The full multiple sequence alignment of all 5 new PALs with the positions homologous to the AvPAL zymophore highlighted. Yellow residues show conservation at these positions whereas blue residues indicate variation.

	SrPAL	BlPAL	DdPAL	MxPAL	PbPAL
SrPAL	**100.00**	56.62	30.27	33.08	30.25
BlPAL	56.62	**100.00**	33.65	32.38	29.62
DdPAL	30.27	33.65	**100.00**	30.69	30.16
MxPAL	33.08	32.38	30.69	**100.00**	45.69
PbPAL	30.25	29.62	30.16	45.69	**100.00**

Figure S6. A percentage identity plot of the new PALs as inferred from the multiple sequence alignment.

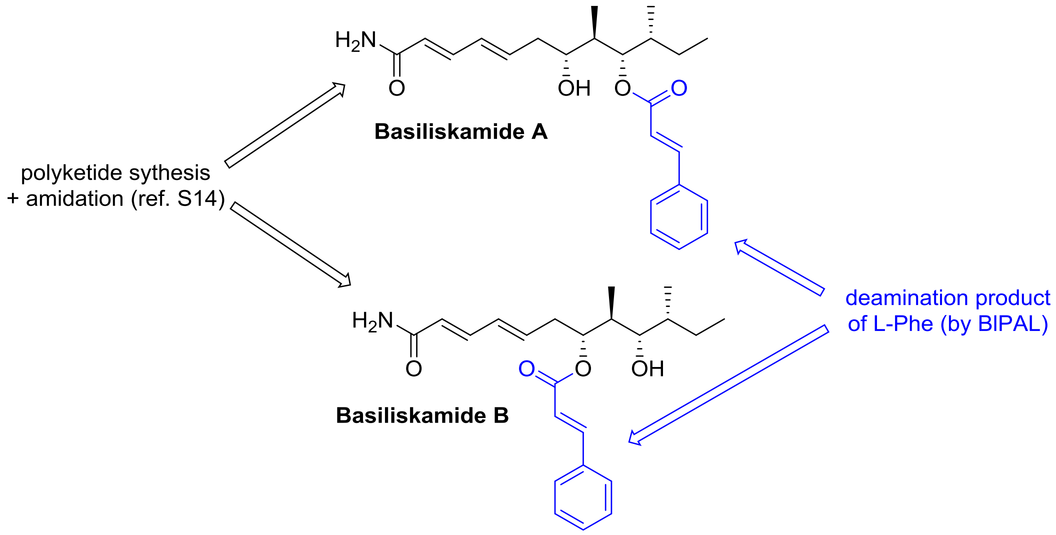

Figure S7. Putative link of BlPAL to the biosynthesis of the basiliskamide antifungal secondary metabolites of *Brevibacillus laterosporus.*[S14]

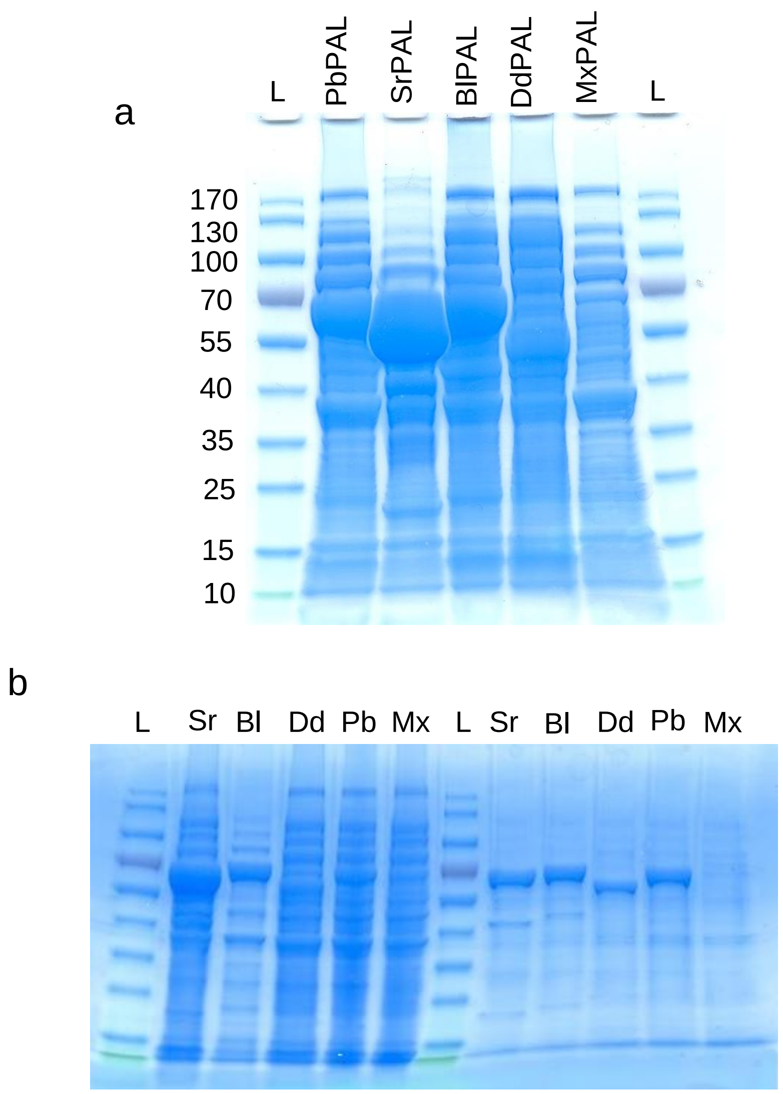

Figure S8. SDS-PAGE analysis of the expression and purification of the new PALs. L = molecular weight marker ladder. (a) whole cell lysates after induction; (b) soluble cell-free extract and purified proteins.

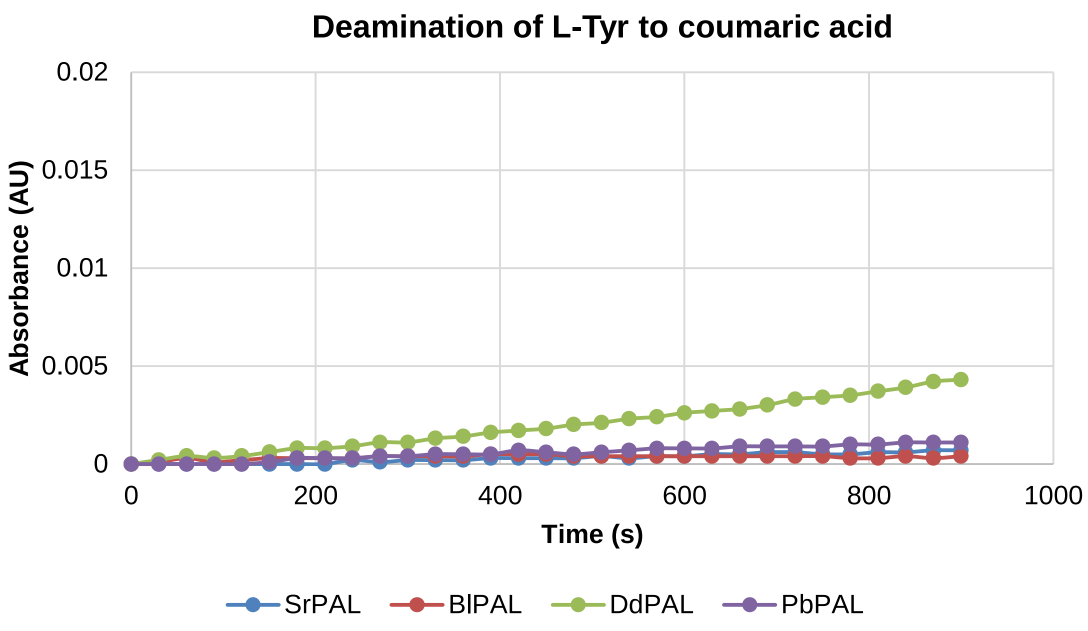

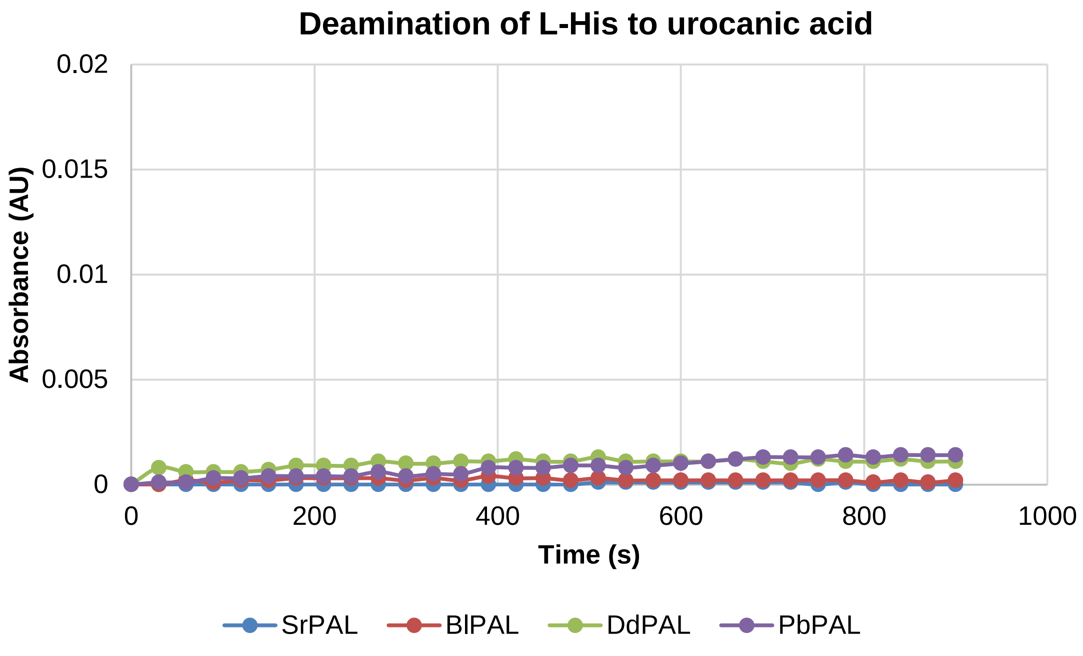

Figure S9. Spectrophotometric assays of TAL and HAL activity with each of the novel ammonia lyases which could be purified for isolated enzyme studies.

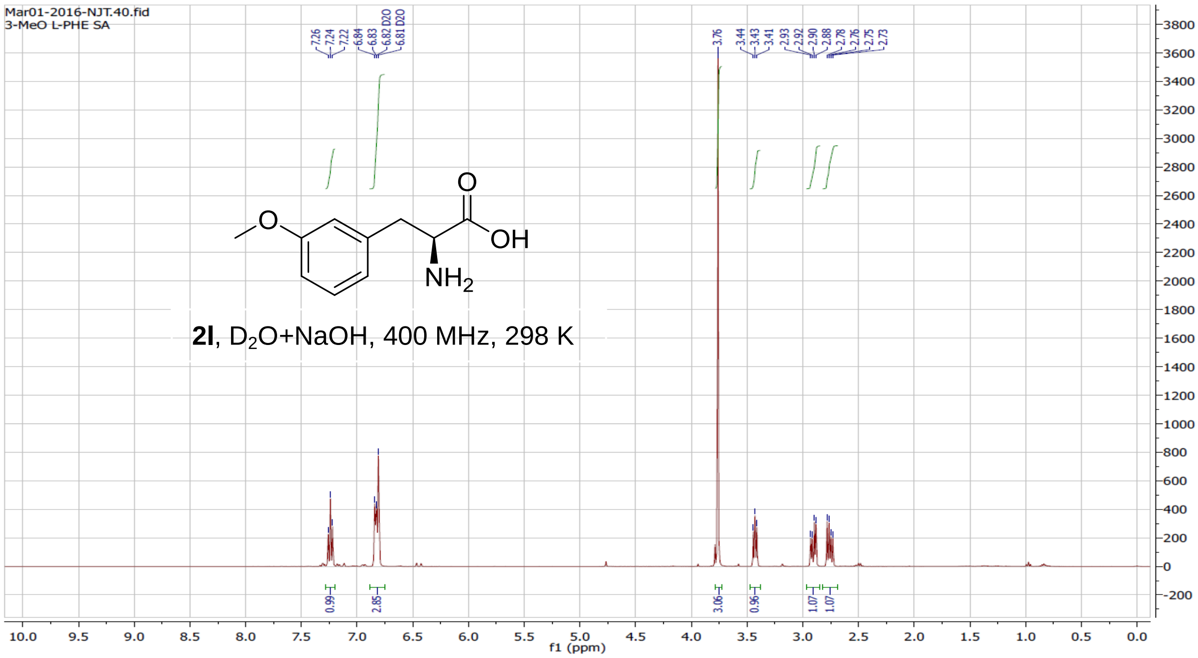

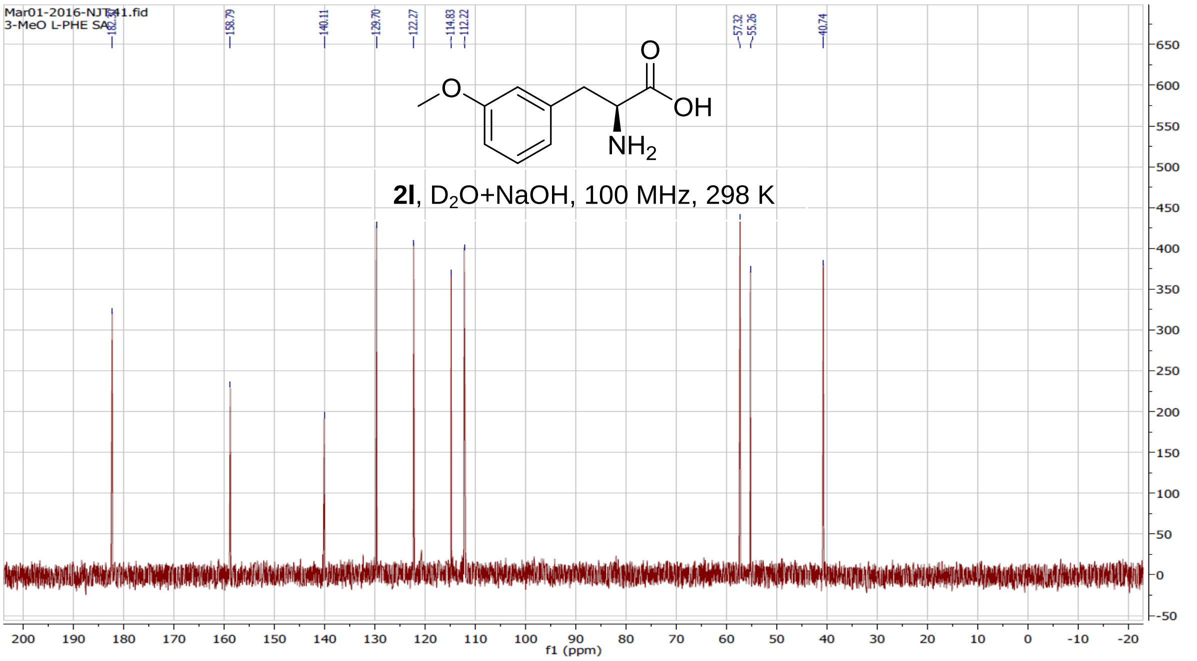

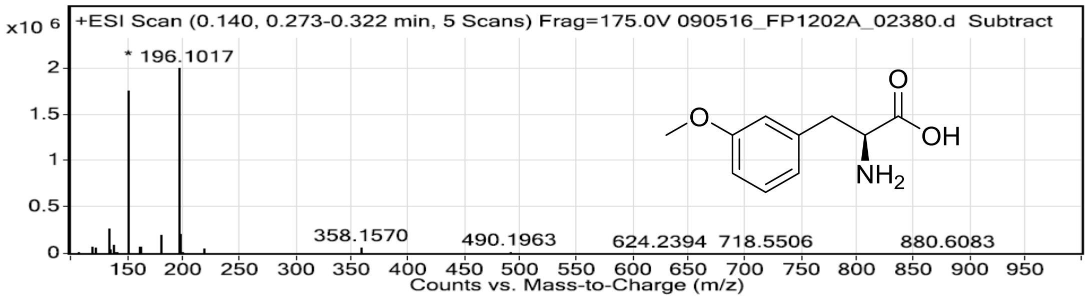

Figure S9. ^{1}H NMR, ^{13}C NMR and HRMS spectra of the (*S*)-**2l** biotransformation product of the reaction of **1l** with PbPAL.

Supplementary Tables

Table S1. Data collection and refinement statistics for crystallographic studies.

	**AvPAL-Y78F-C503S-C565S bound to cinnamate** (PDB ID: 5LTM)
**Data collection**	
Space group	*P* 43 2 2
Cell dimensions	
a, b, c [Å]	78.1, 78.1, 354.5
α, β, γ [°]	90, 90, 90
Wavelength [Å]	0.987
Resolution [Å]*	2.4-29.8 (2.46-2.41)
Rsym or Rmerge*	0.169 (0.047-0.75)
I / σI *	14.9 (3.0)
Completeness [%]*	99.7 (96.6)
CC1/2*	99.7 (85.9)
Redundancy*	12.7 (12.1)
Total observations	552678
Total unique	43577
**Refinement**	
Resolution [Å]	2.41
Rwork / Rfree	0.156 / 0.205
Mean B value [overall Å2]	26.9
r.m.s. deviations	
Bond lengths [Å]	0.009
Bond angles [°]	1.31

* values in parentheses are for highest resolution shell

Table S2. HPLC conditions and retention times for non-chiral analyses.

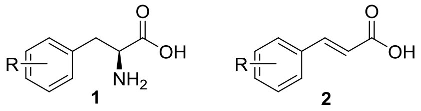

Compound	MeOH [%]	Temp. [°C]	Retention time [min]	
R			**1**	**2**
H	10	40	2.3	5.4
2-F	10	40	2.8	7.2
3-F	10	40	3	7.7
4-F	10	40	2.7	7.1
2-Br	30	40	2.1	4.3
3-Br	30	40	2.8	6.2
4-Br	30	40	2.9	6.5
2-Cl	20	40	3.0	7.4
3-Cl	30	40	2.2	4.8
4-Cl	30	40	2.3	5.0
2-NO_2	10	40	3.9	5.9
3-NO_2	10	40	3.1	6.8
4-NO_2	10	40	2.8	6.1
2-OCH_3	20	40	2.3	4.5
3-OCH_3	20	40	1.9	4.0
4-OCH_3	10	40	2.2	6.4

Table S3. HPLC conditions and retention times for chiral analyses.

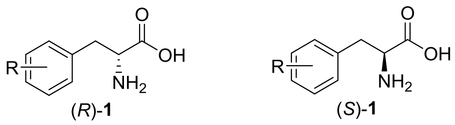

Compound	MeOH [%]	Temp. [°C]	Retention time [min]	
R			**(*R*)-1**	**(*S*)-1**
H	4	25	5.0	6.3
2-F	4	25	5.8	7.5
3-F	4	25	6.5	8.3
4-F	4	25	6.6	8.1
2-Br	14	25	12.1	16.5
3-Br	14	25	19.7	24.8
4-Br	14	25	20.3	26.5
2-Cl	14	25	8.1	10.1
3-Cl	14	25	11.1	15.3
4-Cl	14	25	11.4	14.1
2-NO_2	4	25	6.5	8.1
3-NO_2	4	25	7.4	10.6
4-NO_2	4	25	7.4	8.6
2-OCH_3	4	25	11.3	14.1
3-OCH_3	4	25	13.9	17.1
4-OCH_3	4	25	14.1	17.3

References

S1 M. C. Moffitt, G. V Louie, M. E. Bowman, J. Pence, P. Joseph and B. S. Moore, *Biochemistry*, 2008, **46**, 1004–1012.

S2 W. Szymanski, B. Wu, B. Weiner, S. de Wildeman, B. L. Feringa and D. B. Janssen, *J. Org. Chem.*, 2009, **74**, 9152–9157.

S3 N. J. Weise, F. Parmeggiani, S. T. Ahmed and N. J. Turner, *J. Am. Chem. Soc.*, 2015, **137**, 12977–12983.

S4 S.-X. Huang, J. R. Lohman, T. Huang and B. Shen, *Proc. Natl. Acad. Sci. U. S. A.*, 2013, **110**, 8069–74.

S5 S. Rachid, D. Krug, K. J. Weissman and R. Müller, *J. Biol. Chem.*, 2007, **282**, 21810–21817.

S6 D. Röther, L. Poppe, S. Viergutz, B. Langer and J. Rétey, *Eur. J. Biochem.*, 2001, **268**, 6011–9.

S7 N. A. Magarvey, P. D. Fortin, P. M. Thomas, N. L. Kelleher and C. T. Walsh, *ACS Chem. Biol.*, 2008, **3**, 542–554.

S8 J. S. Williams, M. Thomas and D. J. Clarke, *Microbiology*, 2005, **151**, 2543–50.

S9 L. Feng, U. Wanninayake, S. Strom, J. Geiger and K. D. Walker, *Biochemistry*, 2011, **50**, 2919–2930.

S10 D. Röther, L. Poppe, G. Morlock, S. Viergutz and J. Rétey, *Eur. J. Biochem.*, 2002, **269**, 3065–3075.

S11 J. C. Calabrese, D. B. Jordan, A. Boodhoo, S. Sariaslani and T. Vannelli, *Biochemistry*, 2004, **43**, 11403–16.

S12 Y. Zhu, S. Liao, J. Ye and H. Zhang, *Biotechnol. Lett.*, 2012, **34**, 269–74.

S13 M. Berner, D. Krug and C. Bihlmaier, *J. Bacteriol.*, 2006, **188**, 2666–2673.

S14 C. M. Theodore, B. W. Stamps, J. B. King, L. S. L. Price, D. R. Powell, B. S. Stevenson and R. H. Cichewicz, *PLoS One*, 2014, **9**, e90124.
